# Conservation status and historical relatedness of Italian cattle breeds

**DOI:** 10.1186/s12711-018-0406-x

**Published:** 2018-06-26

**Authors:** Salvatore Mastrangelo, Elena Ciani, Paolo Ajmone Marsan, Alessandro Bagnato, Luca Battaglini, Riccardo Bozzi, Antonello Carta, Gennaro Catillo, Martino Cassandro, Sara Casu, Roberta Ciampolini, Paola Crepaldi, Mariasilvia D’Andrea, Rosalia Di Gerlando, Luca Fontanesi, Maria Longeri, Nicolò P. Macciotta, Roberto Mantovani, Donata Marletta, Donato Matassino, Marcello Mele, Giulio Pagnacco, Camillo Pieramati, Baldassare Portolano, Francesca M. Sarti, Marco Tolone, Fabio Pilla

**Affiliations:** 1 Dipartimento Scienze Agrarie, Alimentari e Forestali, University of Palermo, 90128 Palermo, Italy; 2 Dipartimento di Bioscienze Biotecnologie e Biofarmaceutica, University of Bari, 70124 Bari, Italy; 3 Istituto di Zootecnia, University of Sacro Cuore, 20122 Piacenza, Italy; 4 Dipartimento di Medicina Veterinaria, University of Milano, 20133 Milan, Italy; 5 Dipartimento di Scienze Agrarie Forestali e Alimentari, University of Torino, 10095 Grugliasco, Italy; 6 Dipartimento di Scienze delle Produzioni Agroalimentari e dell’Ambiente, University of Firenze, 50144 Florence, Italy; 7 Unità di Ricerca di Genetica e Biotecnologie, Agris Sardegna, 07100 Sassari, Italy; 8 CREA Research Centre for Animal Production and Acquaculture, CREA, 00015 Monterotondo, Italy; 9 Dipartimento di Agronomia Animali Alimenti Risorse naturali e Ambiente, University of Padova, 35020 Legnaro, Italy; 10 Dipartimento di Scienze Veterinarie, University of Pisa, 56100 Pisa, Italy; 11 Dipartimento Agricoltura, University of Molise, 86100 Campobasso, Italy; 12 Dipartimento di Scienze e tecnologie Agroalimentari, University of Bologna, 40127 Bologna, Italy; 13 Dipartimento di Agraria, University of Sassari, 07100 Sassari, Italy; 14 Dipartimento di Agricoltura, Alimentazione, Ambiente, University of Catania, 95125 Catania, Italy; 15 Divulgazione e Applicazione di Biotecniche Innovative, Consorzio per la Sperimentazione, 82100 Benevento, Italy; 16 Dipartimento di Scienze Agrarie, Alimentari e Agro-ambientali, University of Pisa, 56124 Pisa, Italy; 17 Dipartimento di Medicina Veterinaria, University of Perugia, 06126 Perugia, Italy; 18 Dipartimento di Scienze Agrarie, Alimentari, Ambientali, University of Perugia, 06121 Perugia, Italy; 19 Centro Risorse Bio-Culturali e Sviluppo Locale, University of Molise, 86100 Campobasso, Italy

## Abstract

**Background:**

In the last 50 years, the diversity of cattle breeds has experienced a severe contraction. However, in spite of the growing diffusion of cosmopolite specialized breeds, several local cattle breeds are still farmed in Italy. Genetic characterization of breeds represents an essential step to guide decisions in the management of farm animal genetic resources. The aim of this work was to provide a high-resolution representation of the genome-wide diversity and population structure of Italian local cattle breeds using a medium-density single nucleotide polymorphism (SNP) array.

**Results:**

After quality control filtering, the dataset included 31,013 SNPs for 800 samples from 32 breeds. Our results on the genetic diversity of these breeds agree largely with their recorded history. We observed a low level of genetic diversity, which together with the small size of the effective populations, confirmed that several breeds are threatened with extinction. According to the analysis of runs of homozygosity, evidence of recent inbreeding was strong in some local breeds, such as Garfagnina, Mucca Pisana and Pontremolese. Patterns of genetic differentiation, shared ancestry, admixture events, and the phylogenetic tree, all suggest the presence of gene flow, in particular among breeds that originate from the same geographical area, such as the Sicilian breeds. In spite of the complex admixture events that most Italian cattle breeds have experienced, they have preserved distinctive characteristics and can be clearly discriminated, which is probably due to differences in genetic origin, environment, genetic isolation and inbreeding.

**Conclusions:**

This study is the first exhaustive genome-wide analysis of the diversity of Italian cattle breeds. The results are of significant importance because they will help design and implement conservation strategies. Indeed, efforts to maintain genetic diversity in these breeds are needed. Improvement of systems to record and monitor inbreeding in these breeds may contribute to their in situ conservation and, in view of this, the availability of genomic data is a fundamental resource.

**Electronic supplementary material:**

The online version of this article (10.1186/s12711-018-0406-x) contains supplementary material, which is available to authorized users.

## Background

Domestication of cattle occurred during the Neolithic agricultural revolution, about 8000 years ago, and was associated with dramatic modifications in the socio-economic conditions of most human populations [1]. Cattle became the most relevant domestic species by their ability to supply meat and milk. Moreover, they assumed a role in social and religious ceremonies and games [2]. Artificial and natural selection, coupled with complex evolutionary background scenarios, have led to the creation of a large variety of breeds in terms of phenotypes that are well adapted to a wide range of environments and rearing systems, and to different production purposes [3]. In the last 50 years, the diversity of cattle breeds has experienced a severe contraction, mainly because of the massive worldwide adoption of a few highly productive breeds and intensive selection [4]. As a result, assessing cattle genetic diversity represents an important step in the management of cattle breeding programs [5]. Thanks to the recent advent of high-throughput affordable genotyping techniques, fine genome-wide analysis of the genetic structure and relationships between cattle populations has become possible [3]. These technologies have opened new perspectives to livestock genetics, as part of both the genomic selection revolution in livestock industry and the introduction of genomic approaches in conservation programs for small and endangered populations [6]. In spite of the growing diffusion of the cosmopolite specialized breeds, several local cattle breeds and populations are still farmed in Italy. In the past, local cattle breeds were used as triple purpose animals (work, milk, and meat); then, depending on the region, the animal characteristics, and the geographical boundaries, they began to diverge into the present-day breeds [7]. Nowadays, most of these local breeds are fully adapted to a specific habitat or production system and represent a significant resource to satisfy present and future demands for sustainable farming in a changing environment [8]. Unfortunately, in some cases, only a few purebred representatives of local breeds are available, thus highlighting the need for implementing a national conservation strategy [9]. Towards this aim, detailed information on the genetic diversity and population structure of cattle breeds is needed to guide conservation decisions and the possible use of local cattle populations [4, 8, 10]. The genome-wide genetic diversity and population structure of Italian cattle breeds remain poorly studied compared to local breeds from other European countries, such as France [3], Spain [7, 11], Russia [12] and, in general, of other worldwide cattle breeds [8, 9, 13–15]. Only a few Italian breeds have been characterized using medium-density single nucleotide polymorphism (SNP) arrays [16]. In accordance with the criteria defined by the Italian Breeders Association, there are 16 officially recognized cattle breeds in Italy, i.e. Agerolese, Burlina, Cabannina, Calvana, Cinisara, Garfagnina, Modenese, Modicana, Mucca Pisana, Pezzata Rossa d’Oropa, Pontremolese, Pustertaler, Sarda, Sardo-Bruna, Sardo-Modicana and Ottonese-Varzese. These breeds are characterized by a demographic contraction that, in some cases, is very severe. An official genealogical register is responsible for the safeguard and preservation of these breeds that are not included in any national selection program. Thus, the present study was undertaken to analyze the level of genetic diversity, population structure, admixture patterns and relationships among 30 Italian cattle breeds using medium-density genome-wide SNPs. It represents the first comprehensive genome-wide analysis of Italian cattle diversity. The genomic characterization of these breeds is a first step towards the development of appropriate breeding strategies.

## Methods

### Sample collection and genotyping

Genomic DNA was isolated using a salting-out protocol from buffy coats of nucleated cells that were obtained from whole-blood withdrawn from the jugular vein using EDTA-containing tubes [17]. The DNA concentration was assessed with a NanoDrop ND-1000 spectrophotometer (NanoDrop Technologies, Wilmington, DE).

**Fig. 1:**
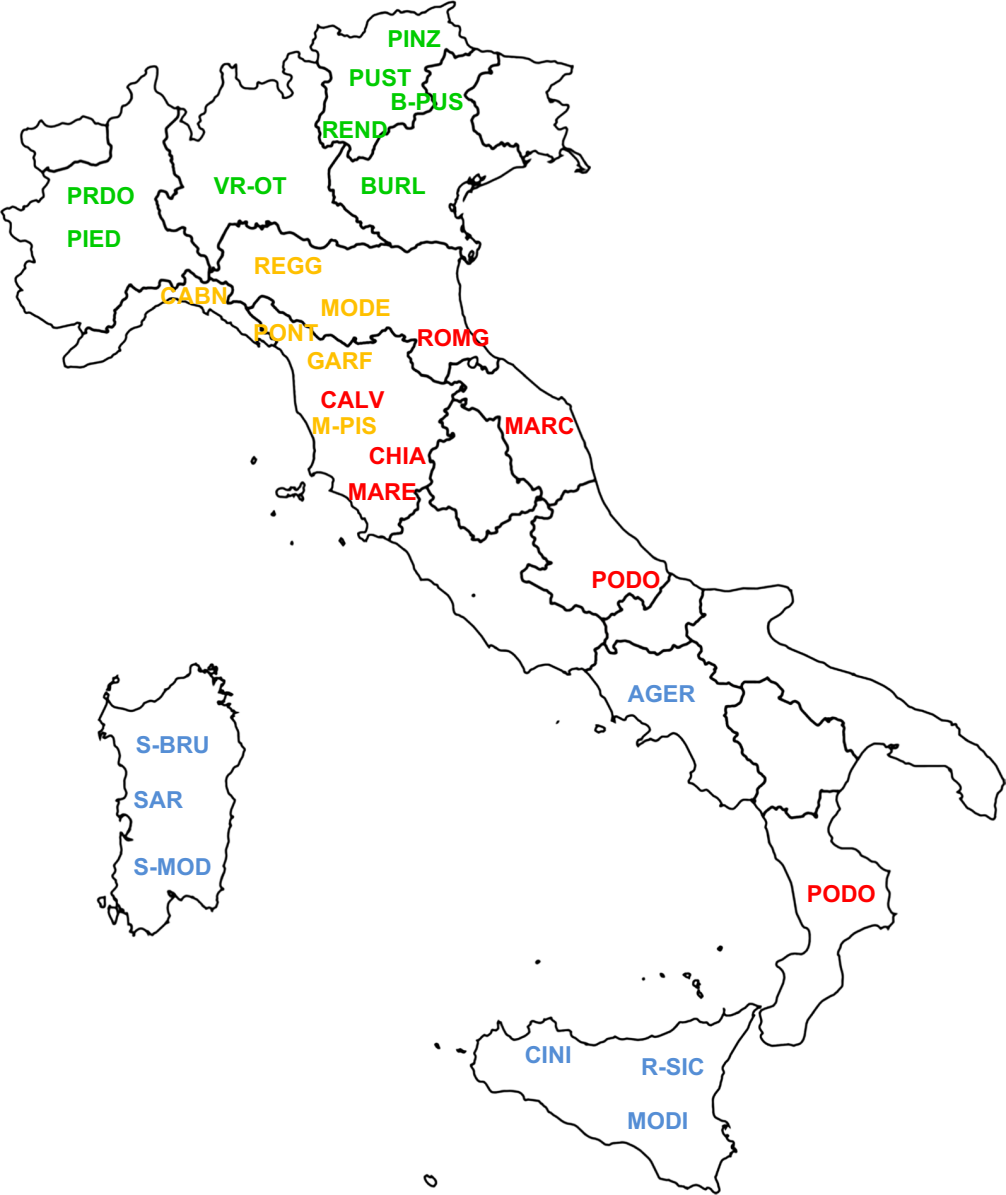
Geographic origin of the analyzed local Italian cattle breeds. Northern (green), Northern-central (orange), Podolian-derived (red) and Southern and islands (blue) breeds. For full definition of breeds (see Additional file 2: Table S1)

A total of 814 animals (10 to 43 per breed) belonging to 30 Italian cattle breeds (Agerolese, Barà-Pustertaler, Burlina, Cabannina, Calvana, Chianina, Cinisara, Garfagnina, Italian Brown, Italian Holstein, Italian Simmental, Marchigiana, Maremmana, Modenese, Modicana, Mucca Pisana, Pezzata Rossa d’Oropa, Piedmontese, Pinzgau, Podolica, Pontremolese, Pustertaler, Reggiana, Rendena, Romagnola, Rossa Siciliana, Sarda, Sardo-Bruna, Sardo-Modicana and Ottonese-Varzese) were selected. In addition, two cosmopolitan breeds reared in Italy (Charolais and Limousin) were included because they are used for cross-breeding with local breeds. Thus, they are relevant to study the genetic relationships between Italian breeds. For a short description of each breed included in this study (see Additional file 1). For sampling representativeness, we selected for each breed unrelated or minimally-related subjects, which were sampled from different farms that cover the usual rearing area. The number of samples and the breed origin of the genotyping data are provided in Additional file 2: Table S1 and the geographical origin of the breeds is illustrated in Fig. 1.

For all animals, genotyping data from the Illumina BovineSNP50 v2 BeadChip array were collected for the analysis. The genotypes of several local breeds were generated within the frame of this study, whereas for some breeds, data were derived in previous studies (see Additional file 2: Table S1). PLINK [18] was used to merge the genotyping data and to conduct quality control tests. In total, 42,561 SNPs were available before filtering. The dataset was filtered to remove animals with more than 10% missing genotypes, and to exclude non-autosomal SNPs, SNPs with a call rate lower than 98% and with a minor allele frequency (MAF) lower than 0.05. After filtering, 31,013 SNPs remained for the analyses on 800 samples from 32 cattle breeds reared in Italy.

### Genetic diversity indices

PLINK [18] was also used to estimate observed (H_o_) and expected (H_e_) heterozygosity, the genomic inbreeding, which is based on the difference between the observed and expected numbers of homozygous genotypes (*F*_HOM_), and average MAF (≥ 0.05). Historical and recent effective population sizes (N_e_) for each breed were estimated with the SNeP package [19], which is based on the relationship between linkage disequilibrium (LD), N_e_ and recombination rate. The contemporary effective population size (cN_e_) was calculated with N_E_ESTIMATOR v. 2 [20], which is based on the random mating model of the LD method [21]. Genotyping data were phased by using the Beagle 3.0.4 package [22], according to Isi-Touru et al. [23]. Expected heterozygosity (H_e__hap) for the haplotypes for each breed was estimated using the R package ADEGENET [24].

### Detection of runs of homozygosity

Runs of homozygosity (ROH) were estimated within each breed using PLINK [17] by applying the following parameters and thresholds to define a ROH: (1) the minimum size of a ROH was set to 40 SNPs; (2) the minimum lengths of a ROH were set to 4, 8 and 16 Mb; (3) a maximum of one SNP with missing genotypes and up to one heterozygous genotype were allowed in a ROH; (4) a minimum density of one SNP per 100 kb; and (5) the maximum gap between consecutive homozygous SNPs was set to 1 Mb. The ROH-based inbreeding coefficient (*F*_ROH_) was calculated for each animal using the method proposed by McQuillan et al. [25], which divides the total length of all ROH in the genome of an individual by the length of the autosomal genome covered by SNPs on the chip (2541.17 Mb). We calculated three genomic coefficients, *F*_ROH>4Mb_, *F*_ROH>8Mb_, and *F*_ROH>16Mb_, based on a minimum ROH length of 4, 8, or 16 Mb, respectively.

### Population genetic structure and admixture

Pairwise genetic relationships were estimated using a matrix of genome-wide identity-by-state (IBS) genetic distances calculated by PLINK [18] and plotted using a multidimensional scaling (MDS) plot. In addition, to investigate more finely the relationships between Italian and European cattle breeds, we also performed a separate MDS analysis by combining our genotyping data with those of 62 European cattle breeds [26]. Population structure was inferred by applying the model-based clustering algorithm implemented in ADMIXTURE [27]. We estimated the most likely number of populations with the cross-validation procedure. We calculated the Reynold’s genetic distances with ARLERQUIN [28] and used them to construct a Neighbor-Net graph with SPLITSTREE [29]. Pairwise estimates of *F*_ST_ were obtained with GENEPOP software [30]. To test the correlations between genetic (*F*_ST_ and Reynold’s genetic distances) and geographical distances between breeds, a Mantel test was performed [24]. We used the coordinates of longitude and latitude of the center of origin for each breed to define the geographical localization (see Additional file 2: Table S1). The geographical distances between each pair of breeds were computed using *distm* function in the R package GEO-SPHERE (http://cran.r-project.org/web/package/geosphere/). Finally, historical relationships and admixture between the considered populations were inferred using the *f3* and *f4* tests implemented in TREEMIX [31], which reconstructs a maximum likelihood tree for the populations based on genome-wide allele frequencies, and then attempts to infer a number of admixture events (edges) to better explain the observed data. The number of admixture events (E) tested ranged from 0 to 10, and the value of E that had the highest log-likelihood was selected as the most predictive model. We then performed the *f3* and *f4* tests implemented in the TREEMIX computer package. In particular, we used the *f3*-statistics (A, B, C) to determine if A was derived from the admixture of populations B and C, and the *f4*-statistics [(A, B) (C, D)] to test the validity of a hierarchical topology in four-population trees.

## Results

### Genetic diversity indices

Genetic diversity parameters are in Table 1. H_o_ and H_e_ ranged from 0.297 ± 0.194 (Pontremolese) to 0.358 ± 0.167 (Piedmontese) and from 0.267 ± 0.187 (Mucca Pisana) to 0.353 ± 0.137 (Sarda), respectively. Mucca Pisana showed the lowest mean MAF (0.200 ± 0.162), whereas the Sarda breed showed the highest mean MAF (0.267 ± 0.139). The highest average *F*_HOM_ were obtained for the Pontremolese (0.195 ± 0.101) and Mucca Pisana (0.183 ± 0.058) breeds, whereas the lowest *F*_HOM_ was obtained for the Sarda breed (0.006 ± 0.063). Estimated H_e_ based on haplotype data (H_e__hap) was higher than that based on genotype data and ranged from 0.323 ± 0.040 (Mucca Pisana) to 0.395 ± 0.003 (Piedmontese), with several Podolian-derived breeds (Calvana, Romagnola, Maremmana and Chianina) having intermediate values (< 0.360). The two H_e_ estimates were correlated (*r *= 0.95, *p* value < 0.001). Estimated N_e_ at *t* generations ago (from 13 to 98) are in Additional file 3: Figure S1. As expected, N_e_ decreased progressively across generations. N_e_ was less than 150 for all breeds at 13 generations ago, except for the Sarda breed (N_e_ = 152). The variation in N_e_ across generations was smallest for Mucca Pisana, Pontremolese and Garfagnina, for which the estimated recent N_e_ (13 generations ago) were less than 40. Ancestral populations of the contemporary Sarda, Cinisara and Podolica breeds exhibited considerably larger N_e_ values, with the largest historical N_e_ values. We also inferred the size of cN_e_ for each breed (Table 1), with Pontremolese and Mucca Pisana having the smallest cN_e_ (less than 10) and Sardo-Bruna the largest cN_e_ (1021). Pearson correlations between genetic diversity indices for SNPs are in Additional file 4: Table S2. As expected, correlations between H_o_, H_e_ and MAF were high (> 0.90, *p* value < 0.001), whereas the correlations between these parameters and cN_e_ were quite low (~ 0.150). Recent and historical N_e_ estimates were not correlated with cN_e_ but moderately correlated with H_o_, H_e_ and MAF (up to 0.810, *p* value < 0.001).

**Table 1:** Genetic diversity indices for the analyzed Italian cattle breeds

Breed	H_o_ ± SD	H_e_ ± SD	H_e__hap ± SD	MAF ± SD	*F*_HOM_ ± SD	cN_e_
Agerolese	0.346 ± 0.176	0.338 ± 0.150	0.372 ± 0.020	0.255 ± 0.145	0.058 ± 0.051	13.6
Bara’-Pustertaler	0.349 ± 0.167	0.342 ± 0.145	0.378 ± 0.021	0.258 ± 0.143	0.051 ± 0.055	31.5
Burlina	0.353 ± 0.169	0.344 ± 0.147	0.377 ± 0.017	0.261 ± 0.143	0.041 ± 0.044	38.0
Cabannina	0.347 ± 0.174	0.336 ± 0.151	0.378 ± 0.013	0.254 ± 0.146	0.056 ± 0.032	35.0
Calvana	0.307 ± 0.198	0.294 ± 0.175	0.334 ± 0.024	0.221 ± 0.158	0.167 ± 0.063	33.5
Charolais	0.353 ± 0.166	0.346 ± 0.144	0.377 ± 0.015	0.262 ± 0.142	0.039 ± 0.066	67.8
Chianina	0.327 ± 0.177	0.323 ± 0.158	0.357 ± 0.016	0.242 ± 0.149	0.111 ± 0.048	118.3
Cinisara	0.343 ± 0.155	0.348 ± 0.141	0.379 ± 0.027	0.263 ± 0.140	0.068 ± 0.070	55.5
Garfagnina	0.312 ± 0.199	0.300 ± 0.177	0.329 ± 0.019	0.226 ± 0.158	0.151 ± 0.053	23.6
Italian Brown	0.307 ± 0.187	0.299 ± 0.171	0.335 ± 0.013	0.223 ± 0.154	0.166 ± 0.033	44.5
Italian Holstein	0.344 ± 0.171	0.338 ± 0.154	0.361 ± 0.014	0.256 ± 0.148	0.064 ± 0.036	60.4
Italian Simmental	0.340 ± 0.168	0.332 ± 0.152	0.368 ± 0.009	0.249 ± 0.145	0.079 ± 0.023	80.7
Limousin	0.345 ± 0.177	0.335 ± 0.152	0.375 ± 0.010	0.253 ± 0.146	0.062 ± 0.024	468.9
Marchigiana	0.339 ± 0.173	0.333 ± 0.151	0.374 ± 0.009	0.250 ± 0.145	0.078 ± 0.023	161.5
Maremmana	0.325 ± 0.192	0.311 ± 0.167	0.357 ± 0.019	0.234 ± 0.154	0.118 ± 0.049	20.3
Modenese	0.341 ± 0.174	0.332 ± 0.153	0.372 ± 0.018	0.251 ± 0.147	0.073 ± 0.045	22.8
Modicana	0.329 ± 0.171	0.328 ± 0.156	0.363 ± 0.025	0.247 ± 0.148	0.105 ± 0.067	69.1
Mucca Pisana	0.301 ± 0.225	0.267 ± 0.187	0.317 ± 0.023	0.200 ± 0.162	0.183 ± 0.058	8.7
Pezzata R. D’oropa	0.333 ± 0.178	0.327 ± 0.158	0.362 ± 0.021	0.246 ± 0.149	0.096 ± 0.054	28.3
Piedmontese	0.358 ± 0.167	0.347 ± 0.141	0.390 ± 0.003	0.262 ± 0.141	0.027 ± 0.011	565.2
Pinzgau	0.349 ± 0.174	0.337 ± 0.151	0.376 ± 0.012	0.254 ± 0.145	0.051 ± 0.030	44.3
Podolica	0.343 ± 0.157	0.349 ± 0.140	0.377 ± 0.029	0.264 ± 0.140	0.066 ± 0.073	110.2
Pontremolese	0.297 ± 0.194	0.292 ± 0.176	0.323 ± 0.040	0.218 ± 0.157	0.195 ± 0.101	7.2
Pustertaler	0.339 ± 0.185	0.323 ± 0.161	0.369 ± 0.011	0.243 ± 0.151	0.078 ± 0.028	26.6
Reggiana	0.346 ± 0.175	0.336 ± 0.150	0.374 ± 0.015	0.253 ± 0.145	0.059 ± 0.040	101.2
Rendena	0.332 ± 0.178	0.325 ± 0.158	0.362 ± 0.010	0.244 ± 0.149	0.096 ± 0.024	527.5
Romagnola	0.325 ± 0.184	0.317 ± 0.163	0.356 ± 0.011	0.238 ± 0.151	0.117 ± 0.026	265.8
Rossa Siciliana	0.356 ± 0.166	0.345 ± 0.143	0.388 ± 0.009	0.261 ± 0.141	0.032 ± 0.023	33.3
Sarda	0.346 ± 0.151	0.353 ± 0.137	0.377 ± 0.025	0.267 ± 0.139	0.060 ± 0.063	62.2
Sardo-Bruna	0.338 ± 0.193	0.334 ± 0.153	0.367 ± 0.034	0.252 ± 0.147	0.082 ± 0.086	1021.3
Sardo-Modicana	0.344 ± 0.168	0.338 ± 0.149	0.378 ± 0.013	0.255 ± 0.145	0.065 ± 0.031	54.8
Varzese-Ottonese	0.351 ± 0.160	0.343 ± 0.145	0.381 ± 0.028	0.259 ± 0.142	0.046 ± 0.071	31.0

Observed (H_o_) and expected (H_e_) heterozygosity, (H_e__hap) expected heterozygosity based on the haplotypes, average minor allele frequency (MAF), inbreeding coefficient (*F*_HOM_), contemporary effective population size (cN_e_) and standard deviation (SD)

### Runs of homozygosity

**Fig. 2:**
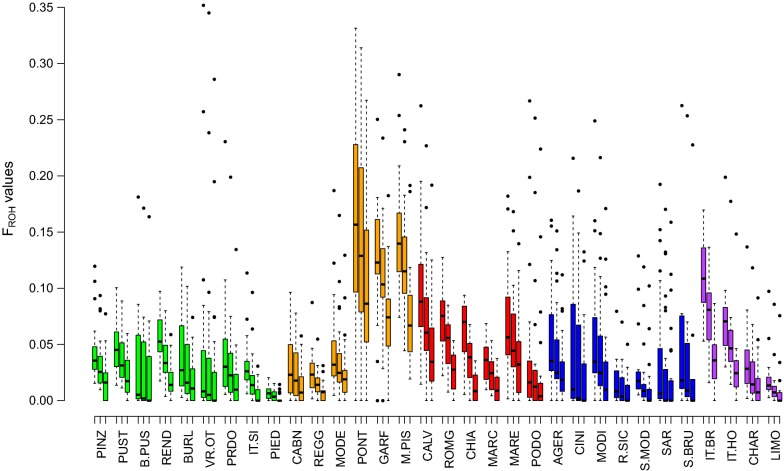
Box plot of the inbreeding coefficients inferred from runs of homozygosity (*F*_ROH_) defined by different minimum ROH lengths (> 4, > 8 and > 16 Mb) for each cattle population according to their geographical distributions. Northern (green), Northern-central (orange), Podolian-derived (red), Southern and islands (blue), and commercial (violet) breeds. For a full definition of breeds (see Additional file 2: Table S1)

Individual genomic inbreeding was evaluated using ROH data. The distributions of the three ROH inbreeding coefficients (*F*_ROH>4 Mb_, *F*_ROH>8 Mb_, and *F*_ROH>16 Mb_) are in Fig. 2. *F*_ROH_ values decreased with increasing minimum length of the ROH. ROH coverage in the genome differed considerably among breeds, with the highest mean values of *F*_ROH_ across all ROH length categories observed for the Garfagnina, Mucca Pisana and Pontremolese breeds. In particular, the Pontremolese breed had more than 10% of the genome covered by ROH in all length categories, and more than 16% by ROH longer than 4 Mb. In contrast, medium and low *F*_ROH_ were found for the other breeds, which is consistent with their larger N_e_ and moderate inbreeding (*F*_HOM_). The breeds with the lowest levels of inbreeding included Piedmontese and Rossa Siciliana. However, the large standard deviation values indicated high variability in autozygosity levels within each breed.

*F*_HOM_ and *F*_ROH>4Mb_ were highly correlated (0.918, *p* value < 0.001) and *F*_ROH>4Mb_ was also negatively correlated with recent and historical N_e_ (~ − 0.74, *p* value < 0.001). The relationship between the number of ROH and the extent of the genome with ROH longer than 4 Mb per individual are in Additional file 5: Figure S2. The patterns of ROH profiles differ among breeds. Most of the animals had between 1 and 20 ROH and less than 200 Mb of their genome were covered by ROH. For several breeds, such as Italian Brown and Mucca Pisana, some animals displayed a larger number of ROH (from 20 to 40) with a total length of 200 to 400 Mb. We also found some extreme animals (Pontremolese, Varzese-Ottonese and Mucca Pisana) with more than 600 Mb of their autosomes covered by ROH, which is equivalent to almost one-fourth of their genome. Differences existed also in the within-breed size. The distribution of the size of ROH also varied within breeds (see Additional file 6: Figure S3) and several individuals had a single ROH longer than 60 Mb.

### Population genetic structure and admixture among Italian breeds

**Fig. 3:**
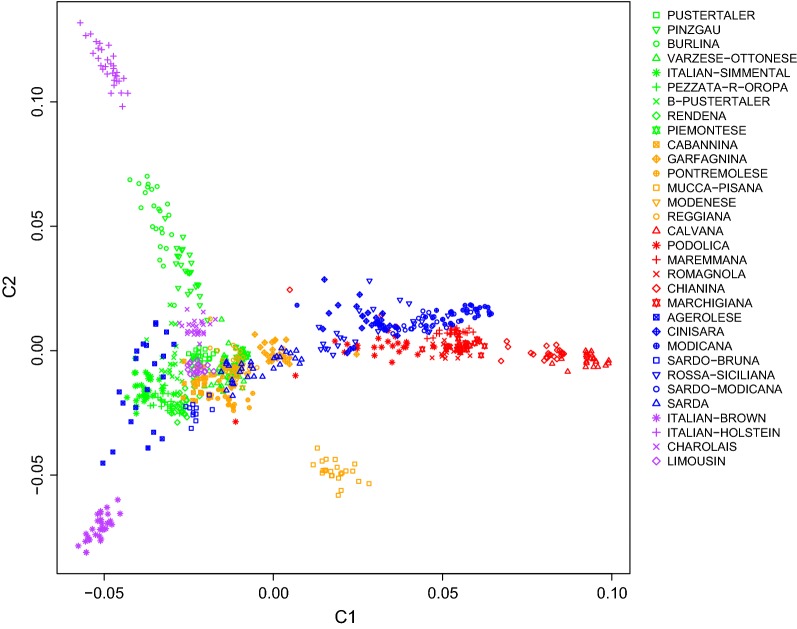
Genetic relationships based on the multidimensional scaling analysis between the analyzed cattle breeds. Points and symbols are colored based on the geographic origin of breeds; the colors are the same as those described in Fig. 2. The first two components, C1 and C2, accounted for 14 and 11%, respectively of the total variation

We used an MDS plot of the pairwise IBS distances to compare the Italian cattle breeds (Fig. 3). The first dimension (C1) distinguished the three Sicilian (Cinisara, Rossa Siciliana, Modicana) and the Podolian-derived breeds (Romagnola, Marchigiana, Chianina, Calvana, Maremmana and Podolica) from the other breeds (right side of the plot). Moreover, the Sardo-Modicana breed was positioned in the same area. The Northern and Northern-central Italian populations formed a distinct group, which was clearly separated from the Sicilian and the Podolian-derived breeds on the first axis. The Burlina and Pinzgau were genetically differentiated and positioned a little further from the breeds from Northern Italy and near the Italian Holstein (at the top left of the plot), while the other breeds (Piedmontese, Pustertaler, Barà-Pustertaler, Rendena, Pezzata Rossa d’Oropa, Reggiana, Modenese, Varzese-Ottonese, Cabannina and Pontremolese), overlapped in a single cluster. This cluster also included the Sardo-Bruna, Italian Simmental and the two commercial meat breeds. The Garfagnina appeared as a distinct cluster and was positioned between the two principal clusters. Some Sarda animals clustered with Garfagnina, whereas others were mixed with the Northern and Northern-central Italian populations, and in particular with Piedmontese. The results also indicated that Mucca Pisana was isolated from the other breeds reared in Tuscany. The second dimension C2 clearly separated two other non-overlapping breeds, Italian Holstein and Italian Brown. Finally, among all the analyzed breeds, Agerolese showed a more spread out cluster, with some animals positioned near the Italian Simmental and Italian Brown breeds.

**Fig. 4:**
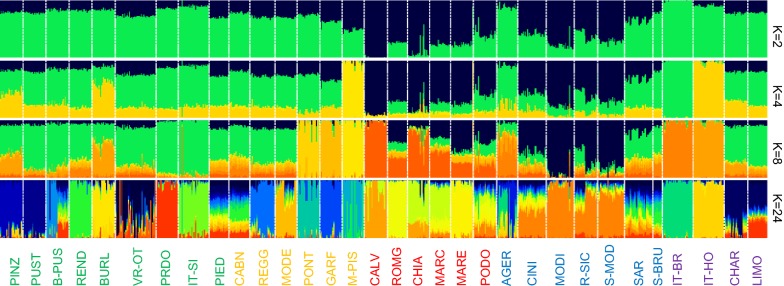
Model-based clustering of the estimated membership fractions of individuals from the 32 breeds analyzed in each of the K inferred clusters (K = 2, 4, 8 and 24). Names of breeds are colored according to their geographical distributions as described in Fig. 2. For a full definition of breeds (see Additional file 2: Table S1)

We tested admixture among breeds and groups of breeds by model-based clustering. The ADMIXTURE plots showed the results for K ranging from 2 to 32 (Fig. 4 and see Additional file 7: Figure S4). The first split (K = 2) differentiated Calvana and Italian Brown from all other breeds. Additional breed-specific clusters were observed at K = 4, i.e. Mucca Pisana and Italian Holstein, and at K = 8, i.e. Italian Simmental, Garfagnina, Pontremolese and Modicana (Fig. 4).

**Fig. 5:**
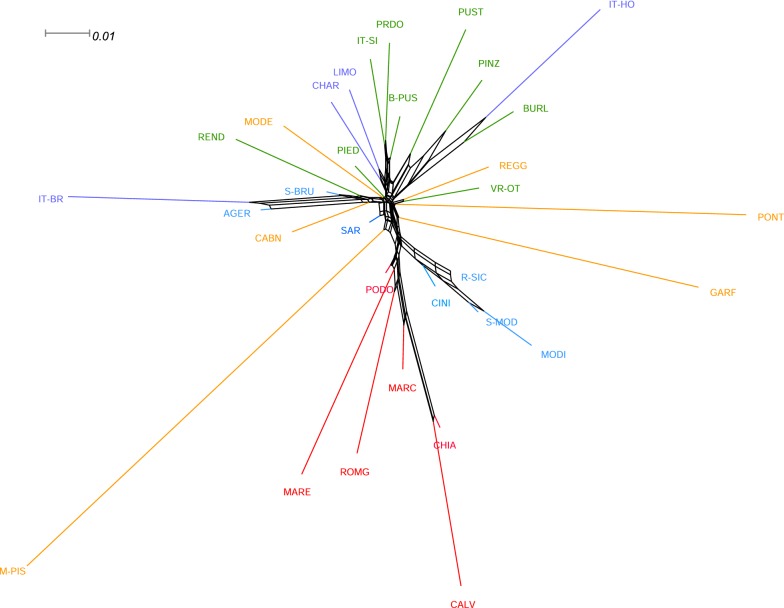
Relationship between breeds based on the Reynold’s genetic distance. An allele frequency-dependent distance metric (Reynolds) was used to construct a Neighbor-Net graph that relates the breeds. Names of breeds are colored according to their geographical distributions as described in Fig. 2. For a full definition of breeds (see Additional file 2: Table S1)

When K increased from 8 to 32, breeds were progressively assigned to separate clusters: Rendena and Maremmana at K = 12, Romagnola and Pustertaler at K = 16, Pinzgau and Burlina at K = 20 (see Additional file 7: Figure S4). The most probable number of populations present in the total sample, as suggested by the ADMIXTURE cross-validation procedure was K = 24 (see Additional file 8: Figure S5), since at this value, each breed formed a distinct cluster although there was some variation. In fact, several breeds (Barà-Pustertaler, Varzese-Ottonese, Piedmontese, Cabannina, Podolica, Agerolese, Cinisara, Rossa Siciliana, Sarda, Sardo-Bruna, and Limousin) showed less distinct clusters. The same trend was also observed at K = 28 and 32 (see Additional file 7: Figure S4). The Neighbor-Net graph, which was constructed based on Reynold’s genetic distances between pairs of breeds, showed another picture of the genetic relationships among the analyzed breeds (Fig. 5). Consistent with the MDS plot, the Neighbor-Net graph shows several clear clusters and relationships between breeds, i.e. Italian Brown, Agerolese and Sardo-Bruna; Podolian-derived breeds; Sicilian breeds and Sardo-Modicana; and Burlina, Pinzgau, Pustertaler and Italian Holstein formed clear sub-branches. The shortest and longest branches were observed for Sarda and Mucca Pisana, respectively, which is consistent with the genetic diversity results (Table 1).

Details of the level of pairwise genetic differentiation are in Additional file 9: Table S3. Based on the pairwise *F*_ST_ among all the populations, Mucca Pisana was again the most divergent breed. In general, for some breeds, *F*_ST_ was not found to be a proxy for geographic distance. For example, *F*_ST_ was highest between two Tuscany breeds, Mucca Pisana and Pontremolese, (*F*_ST_ = 0.222) and lowest between Sarda and Sardo-Bruna (*F*_ST_ = 0.016). To test the correlations between genetic (*F*_ST_ and Reynold’s genetic distances) and geographical distances, we performed a Mantel test. The results showed no concordance between *F*_ST_ and geographical distances among all breeds (*r* = − 0.073, *p* value = 0.75) (see Additional file 10: Figure S6), even after removing the commercial breeds (Charolais, Limousin, Italian Brown, Italian Holstein and Simmental) (*r* = − 0.160, *p* value = 0.94). When only the Sicilian breeds were considered, the correlation increased but remained statistically non-significant (*r* = 0.979, *p* value = 0.33). We also conducted a Mantel test between Reynold’s genetic distances and geographical distances among breeds but again, no correlation was observed (*r* = − 0.078, *p* value = 0.78).

**Fig. 6:**
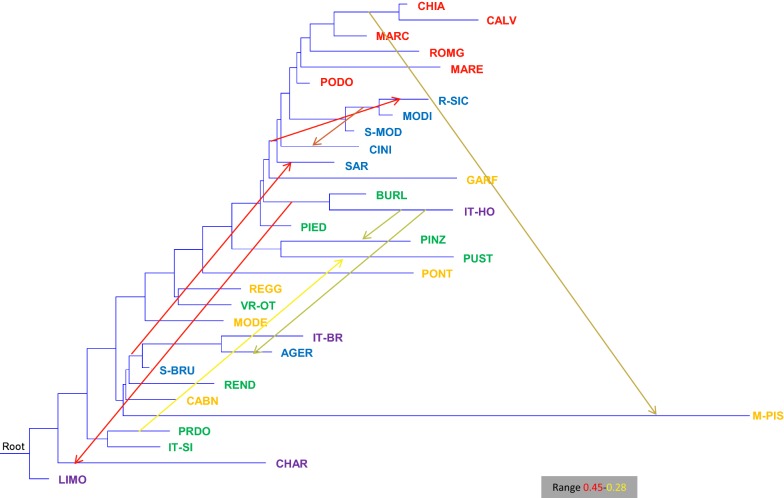
Maximum likelihood tree inferred from 32 cattle breeds when eight migration events are allowed. Migration arrows are colored according to their weight. Name of breeds are colored according to their geographical distributions as described in Fig. 2. For a full definition of breeds (see Additional file 2: Table S1)

The TREEMIX results highlighted several admixture events (Fig. 6) with most of them being expected based on the history of the breeds, such as admixture between Italian Holstein and Agerolese, between the group Calvana/Chianina and Mucca Pisana, between Italian Holstein and Pinzgau, between Pezzata Rossa d’Oropa and Pustertaler, between the group Agerolese/Italian Brown/Sardo-Bruna and Sarda, between the group Modicana/Rossa Siciliana and Cinisara, between Pinzgau and Pustertaler. Some admixture events were less obvious, e.g. between the Italian Holstein/Burlina and Charolais group, between the Italian Simmental/Pezzata Rossa d’Oropa group and the Rendena/Piedmontese/Charolais group. Finally, we detected a basal admixture event that involved several breeds from Central and Southern Italy (Chianina, Calvana, Marchigiana, Romagnola, Maremmana and Podolica) and from Sicily and Sardinia (Sardo-Modicana, Modicana, Rossa Siciliana, Cinisara and Sarda). Results from the *f3* test highlighted clear signs of admixture between Rossa Siciliana and Modicana (see Additional file 11: Table S4). The admixed nature of Rossa Siciliana was also supported by the *f4* test (see Additional file 12: Table S5) that highlighted a clear gene flow with Modicana, Sardo-Modicana and Cinisara (significant negative Z values) and with Limousin (significant positive Z values).

### Relationships between European cattle breeds

To investigate the genetic relationships between Italian and European cattle breeds, we performed an MDS analysis using a combined dataset that included 32,115 SNPs and 1437 individuals (see Additional file 13: Figure S7). The second dimension C2 differentiated the Sicilian and Podolian-derived breeds from all other Italian breeds. Turkish breeds cluster with Sicilian and Podolian-derived breeds and Northern and Northern-central Italian populations were much closer to several French, German and Switzerland breeds. The Spanish breeds were positioned between the Northern/Northern-central and the Sicilian/Podolian-derived breeds. The first dimension C1 separated all the aforementioned breeds from the other European breeds included in this study.

## Discussion

Improving our knowledge about the within-breed diversity and the between-breed relationships and structure in cattle is fundamental for improving selection designs and breeds, understanding environmental adaptation, enhancing efficient use of the breeds and implementing conservation programs [32, 33]. With the development of high-throughput genotyping technologies, analyzing the genetic structure of livestock species has become feasible. However, to date, much of the effort has been devoted to dominant commercial breeds, with local breeds generally poorly studied [34], although they can represent outstanding genetic resources for the local economy. This study investigated the genome-wide structure of 30 Italian local cattle and two cosmopolitan breeds using medium-density genome-wide SNPs. Except for a very few breeds (e.g. Valdostana), we were able to sample all the local cattle breeds reared in Italy and officially recognized. Therefore, our analysis concerns the largest and most complete dataset available for Italian cattle breeds.

### Genetic diversity

Although homozygosity may be overestimated for populations that were not included in the design of the SNP array (ascertainment bias) [13], the Illumina set of 50 K SNPs was highly informative for all the Italian cattle breeds analyzed here. Heterozygosity values, polymorphism levels and recent and historical N_e_ estimates were very consistent. Average MAF were homogeneous among the breeds, and, on average, SNPs were equally informative for all breeds, which is consistent with previous observations [13], even if at the individual level, the MAF of SNPs can vary considerably. The H_e_ and MAF values obtained in our study agree with the range of values that was reported in a study on the development and characterization of a medium-density SNP genotyping assay for cattle [13], and are similar to those observed in other European breeds [3] using the same SNP genotyping assay. This finding is likely due to the effect of the European ancestry of Italian cattle breeds [3]. We observed a decline in N_e_ with time in all breeds, as previously reported for other cattle breeds [4, 12, 23]. In fact, estimated trends in N_e_ indicate that, in the past, the N_e_ of Italian cattle breeds was large. The size of cN_e_ differed among breeds, and several Italian breeds displayed small values, which is consistent with their low genetic diversity and high genomic inbreeding coefficients. In agreement with our results, small cN_e_ were reported also for Iranian native cattle breeds [35] (from 13 to 107) and the Belgian Campine breed [36]. In the last 50 years, the number of individuals of these latter breeds has decreased dramatically, as is the case for Italian local breeds. With such small cN_e_, their inbreeding rate has increased markedly until recently, as shown by the detection of long ROH in several local Italian breeds. The small cN_e_ inferred for most of the Italian local breeds, as well as the low values of their genetic diversity indices, may be also a consequence of population bottlenecks that occurred due to the geographic isolation of some farms and the reduced interest of breeders for these breeds [37]. In contrast, the Sarda breed had a larger N_e_ at all generations, and the Sardo-Bruna had the largest cN_e_, which is probably due to admixture within these two breeds and crossbreeding with other cattle breeds. In general, strong selection pressures and use of artificial insemination (AI) are the main causes of small N_e_ in livestock. In local cattle breeds, selection pressure is usually very weak and AI is almost absent, but conversely, uncontrolled mating of related animals is common, and thus inbreeding and low genetic diversity are the most likely cause of their small N_e_ [37]. No correlation was found between cN_e_ and recent and historical N_e_ estimated 13, 20 and 80 generations ago. Estimates of N_e_ vary a lot with the method used [38]. Moreover, it should be emphasized that these estimates can be strongly biased when the sample size is small, probably because of the LD generated by the sampling process [33]. Actually, none of the formulas proposed to date for estimating N_e_ from LD provide reliable predictions [39], probably because they all rely on simplifications or assumptions. Therefore, these estimates (in particular the cN_e_) must be considered with care, even more so when the sample size is less than 15 animals [35]. Anyway, our results are in agreement with other studies in cattle [4, 23].

Across all the breeds analyzed here, generally, the cosmopolitan breeds (Italian Holstein, Brown and Simmental, Limousin and Charolais) showed moderate levels of genetic diversity. Moreover, although some breeds (Rossa Siciliana, Sardo-Bruna, Sardo-Modicana, Agerolese) are endangered or have a small census population size, they did not show signals of low levels of genetic diversity. ROH analysis and N_e_ values corroborated these results, likely because of their admixed origins. However, the levels of polymorphism and genetic variability were lower in Pontremolese and Mucca Pisana than in all other breeds. These results are as expected since these two breeds experienced a stronger reduction in numerical size in the second half of the 20th century. Currently, the Pontremolese population includes less than 30 cows and the Mucca Pisana population is composed of nearly 200 cows. In both cases, the number of bulls is very small (2 and 10 for Pontremolese and Mucca Pisana, respectively). The low genetic variability observed for these breeds agrees with the theoretical expectation for populations, which have undergone a severe bottleneck [40]. Genetic diversity is an intrinsic factor that influences the adaptive capacity and resilience of populations. This low genome-wide genetic variability in these two breeds, as that observed in other local breeds, may be also related with a lower adaptation potential, which could represent a threat to their long-term persistence [33]. Therefore, our findings raise the possibility of a risk for the genetic diversity of Italian local cattle breeds, and the decrease in N_e_ should be taken into account and monitored.

### Runs of homozygosity

Abundant genome-wide SNPs are particularly suitable for detecting genomic regions with reduced heterozygosity and, recently, an alternative method called runs of homozygosity (ROH) was implemented [41]. Currently, ROH-based *F* estimates (*F*_ROH_) are considered one of the most powerful approaches to detect inbreeding effects [42] and may also reveal recent population bottlenecks or signatures of directional selection [25, 43]. Because the parameters used to detect ROH vary among analyses, it is not easy to compare results from different studies on ROH. Moreover, ROH have rarely been estimated in local breeds. Notwithstanding, our results are in agreement with the values reported in other studies on cattle [4, 44–46]. Analysis of individual ROH may be useful for conservation programs, since animals that have high levels of ROH, as observed in some Pontremolese, Varzese-Ottonese and Mucca Pisana animals, could be excluded or assigned lower priority for mating purposes in endangered populations, to minimize the loss in genetic diversity and maintain or increase N_e_ [47]. The length of ROH represents an important source of information on past and present demographic and genetic processes that shape the genetic diversity of livestock species [48]. Since recombination events split long chromosome segments, long ROH are a consequence of recent inbreeding. In contrast, short ROH are indicative of relatedness dating back to more ancient times [49], which are in most cases not considered in an individual’s recorded pedigree. Thus, under the assumption that 1 cM = 1 Mb, a minimum ROH length of 4, 8 or 16 Mb implies a common ancestor 12, 6 or 3 generations ago, respectively [6]. Thus, analyses that are carried out with ROH of different lengths allow us to estimate the distance between the current and base populations, and provide information on the age of inbreeding [4]. Generally, all breeds showed long ROH, but for some local breeds (such as Varzese-Ottonese, Calvana, Pontremolese, Mucca Pisana) their number increased. These results are also corroborated by the higher *F*_ROH>16Mb_ obtained for Garfagnina, Mucca Pisana and Pontremolese. Therefore, strong evidence of recent inbreeding (3 generations ago) exists for these Italian breeds, due to the decrease in their N_e_. In fact, isolation of breeds with a small population size increases the probability that identical segments are inherited. It is likely that the long ROH are also signatures of the extensive use of a few bulls within herds and of mating among relatives. If long ROH accumulate in the genome of some individuals, they could seriously impact the overall biological fitness [50]. Indeed, long ROH can be enriched in genomic regions that carry deleterious mutations, and Kim et al. [51] showed that a strong relationship exists between the proportion of ROH in the genome and the number of individuals that carry deleterious homozygous mutations. Hence, the ROH levels estimated in our study may be informative to better understand the inbreeding history of the breeds analyzed. For example, this consideration is consistent with the management strategies known for the Pontremolese breed and its demographic decline that was reported ~ 30 years ago [52]. Similar results were also reported in Spanish goats, in which the most abundant and long ROH were identified in breeds that are at the verge of extinction [50]. On the contrary, the high *F*_ROH_ and low N_e_ values observed for the Italian Brown breed can be ascribed to the intense use of a small number of (closely related) bulls for artificial insemination. More generally, these results highlight that both ancient and recent inbreeding have impacted the genome of Italian cattle breeds and that several local breeds, in particular the four autochthonous cattle breeds of Tuscany (Pontremolese, Garfagnina, Mucca Pisana and Maremmana), have probably not undergone recent extensive crossbreeding since the long ROH in their genomes have not been broken down [47]. This scenario probably reflects practices in the management of breeds with less controlled breeding that do not always prevent crosses between related animals, although they deserve special attention and conservation efforts. On the contrary, the limited occurrence of long ROH in Piedmontese indicates that this breed has benefited from proper breed management and has a sufficiently large N_e_ and thus a low degree of recent consanguinity. Therefore, we conclude that the genetic diversity indices, the effective population size and the genomic inbreeding levels were congruent with the protection status of the local Italian cattle breeds based on their reduced demographic size. The results also reflect the need to implement conservation programs, in particular for the breeds with a limited distribution.

### Population structure and admixture

Determination of the structure and the genetic relationships of a population has proven useful in conservation programs and for developing suitable management practices [4, 15, 32–35]. To understand these aspects, we carried out an MDS and ADMIXTURE analysis, inferred TREEMIX ancestry graphs, calculated the Reynold’s genetic distances and the pairwise estimates of *F*_ST_ for the Italian local cattle breeds. Except for Podolian-derived breeds, the MDS grossly separated the breeds according to their genetic origin and/or to their geographical proximity between breeding areas. A previous study on breeds (Calvana, Chianina and Maremmana) reared in the ancient Etruria region (Tuscany, Central Italy), reported that they are genetically closer to Near Eastern than to European genetic stocks [53]. This oriental genetic signature was observed also in modern Tuscan human populations, which have been shown to be genetically close to Anatolian and Middle Eastern human populations [53]. To better understand the genetic relationships between Italian cattle breeds, by considering the possible connections with breeds/populations that are presumed to have contributed to the shaping of the current genetic background of some of them, we performed an additional MDS analysis among Italian and European cattle breeds. Clustering of the breeds is generally consistent with their geographical origin. The Northern Italian breeds are much closer to several European breeds than to other Italian breeds, which indicates a contribution of continental European ancestry in the formation of these Italian cattle breeds. Another observation was that, even on the European scale, populations of the same major commercial breeds cluster together, e.g. Italian Holstein and European Holstein, and Italian Brown and Brown Swiss. Moreover, our results are consistent with those of a mtDNA study [53], which showed that the Turkish breeds (such as the Anatolian Black breed), clustered with the Tuscany (particularly with Calvana and Chianina) and the Cinisara breeds. The observed separation between Podolian-derived breeds and Northern Italian populations corroborates previous studies on the genetic relationship of some local Italian cattle breeds using blood group systems and blood proteins [54]. Our results were also corroborated by a recent study on mtDNA variation in different Podolian breeds [55], which revealed a genetic proximity between the Italian Podolian-derived and the Turkish breeds. Their findings also show that, generally, the values of haplotype diversity indices were lower in some Italian Podolian-derived (Calvana, Podolica and Maremmana) than in non-Podolian breeds, which is consistent with our results of H_e__hap.

The presence of a general North–South distribution of the genetic diversity along the Italian Peninsula was highlighted and confirmed by the low genetic differentiation (*F*_ST_) among some local breeds from the same geographic area, such as between Sarda and Sardo-Bruna or among the Sicilian breeds. Previously, a similar geographical pattern was described by using a medium-density SNP array in Italian goat [56] and sheep breeds [57]. Our results did not show any obvious relationship between the patterns of clustering and the productive aptitude of Italian cattle breeds, contrary to what was reported for sheep breeds [57]. Moreover, no significant correlation between genetic differentiation and geographical distances was observed for Italian cattle breeds. Traspov et al. [58] showed that, in local pig breeds, there was no geographical gradient of the distribution of their genetic variability and suggested that, even for breeds that had close geographical origins (as was the case for the Mucca Pisana and Pontremolese breeds in our study), the implemented breeding schemes led to a high genetic differentiation. Moreover, similar results were also observed in pig populations from America [59], which are probably the consequence of complex genetic histories. Therefore, these results confirm that the admixture between geographically distant populations could be a major force in breaking regional genetic-geography concordance [60]. Although the Northern and Northern-central Italian populations have different demographic histories and different breeding goals, our results show that they overlap in a cluster and they cannot be easily discriminated; furthermore, their MDS coordinates identified only small areas on the plot, as a consequence of the reduced within-breed genetic variability [56]. In general, the MDS plot was consistent with the admixture analysis, in which some kind of hierarchical structuring was identified. The plot obtained by ADMIXTURE showed that at K = 8, some groups shared a substantial proportion of their ancestry, such as the Sicilian (Cinisara, Modicana and Rossa Siciliana), the Podolian-derived breeds and Northern and Northern-central Italian populations. This observation was also consistent with the TREEMIX results. It is worth mentioning that, on the one hand, the breeds that were the most homogeneous at the lower K values also displayed the lowest heterozygosity level, a phenomenon known as ‘inbreeding bias’ [61]. On the other hand, for the breeds that, at the best K value (24), displayed less distinct clusters, there was no evidence for high levels of inbreeding due to their admixed origins, and this suggests that crossbreeding with other breeds occurred. Therefore, our results are largely consistent with the breeding history of the Italian cattle breeds, given that some breeds are the result of crossbreeding. For example, the Sardo-Modicana breed originated by crossing local Sarda cows with Modicana bulls [54] and the Sardo-Bruna breed by crossing local Sarda cows with Brown bulls. The genetic relationships between the Chianina and Calvana breeds are strongly supported by historical data and were previously investigated through AFLP (amplified fragment length polymorphism) [62] and microsatellite markers [63]. Some Sarda animals were mixed with Piedmontese, which reflects possible admixture. Similarly, Rossa Siciliana showed a high level of gene flow with Limousin. In fact, crossbreeding between local breeds and meat breeds is common practice, to improve meat production but also for long-term breeding. Therefore, this practice explains the results on admixture between local and meat breeds. In addition, the genetic structure of the Agerolese breed was typical of a breed that showed admixture with other breeds, as confirmed by admixture events in Italian Holstein and Italian Brown, and in agreement with its genetic origins. It is indeed a dual-purpose breed, which originated during the 19th century from autochthonous cows crossed with Brown Swiss and Holstein–Friesian bulls [64]. However, the limited number of males (e.g. only 13 males in natural service in the 2002) [64] that were available in the breeding program could result in a limited N_e_ size that can, in turn, lead to a strong impact of genetic drift. Our results also indicate that the genetic component of some commercial dairy breeds, such as the Italian Holstein, was relatively small in the Italian local breeds. In fact, the lowest *F*_ST_ values of the Italian Holstein was with the Burlina breed (0.066). Moreover, as illustrated by the MDS and ADMIXTURE results, the Italian Holstein breed is genetically distant from the local breeds. Nonetheless, differentiation between some local breeds (Barà-Pustertaler, Sarda, and Sardo-Bruna) and the commercial meat breeds (Limousin and Charolais) was very low (*F*_ST_ ~ 0.04), which suggests, as mentioned above, that the use of these breeds could have affected their genetic background. Among all the local breeds, Mucca Pisana was highly differentiated and presented only low levels of admixture with other breeds; this breed consistently demonstrated higher *F*_ST_ values with the other breeds analyzed. In a recent study on Russian cattle breeds, analogous results were reported for the Yakut cattle, which was the most divergent breed [12]. For both Yakut and Mucca Pisana breeds, it is not clear why they are so divergent. It may be due to a combination of a small historical population size and a long history of isolation from other breeds. Our results also suggested that, compared to other breeds, the genome of the Mucca Pisana breed contains less genetic data from any other ancestral breed that it may have interacted with, which can be considered as a typical signal of inbreeding [65] and is consistent with the values of the genetic diversity indices. The long branch observed for this breed suggests that it is a differentiated and isolated population with a small N_e_ size. In fact, Mucca Pisana showed the smallest recent and past N_e_ estimates. In a study on sheep breeds, Kijas et al. [66] found that short branches were associated mainly to highly heterozygous breeds, while long branches were associated to much less heterozygous breeds. The above evidence for the Mucca Pisana breed was also confirmed by the MDS, ADMIXTURE, TREEMIX and *F*_ST_ analyses, and can be explained by its demographic history since it was reared for a long time in a geographically separate valley (Valle del Serchio) [67]. Therefore, it is likely that this breed experienced reproductive isolation and reduced gene flow, and thus acquired a strong genetic identity. Since the Neighbor-Network analysis takes gene flow among breeds into consideration (reticulation), it may provide a more likely reconstruction compared to linear representations [65]. Indeed the Neighbor-Network graph highlighted several clear clusters and relationships between breeds that originated from the same area, as shown in the MDS plot (Fig. 3). The sub-branches of the Neighbor-Network graph were also in agreement with the results of the admixture, genetic relationship and genetic diversity analyses within breeds. In fact, the length of each sub-branch reflected the results of recent and past N_e_ estimates. The reticulations towards the extremity of the networks also suggest increased genetic relatedness between breeds and therefore past hybridization events between these populations, and are consistent with several of the admixture events highlighted by TREEMIX between breeds and groups. Indeed, Neighbor-Net is a robust tool for reconstructing phylogenetic networks. Therefore, the low pairwise *F*_st_ values, shared ancestry, admixture events, and reticulations observed on the phylogenetic tree between some breeds, as well as between the Sicilian breeds, all suggest high levels of gene flow between these populations, and as well between some breeds originating from the same area. These results can be attributed to the fact that geographical proximity facilitates the gene flow, and that breeds from the same breeding area are more likely to have common ancestries.

## Conclusions

Our study represents the first comprehensive analysis of the genomic diversity and population structure of Italian local cattle breeds. All the analyses revealed genetic relationships, gene flow and admixture events for several Italian cattle breeds. However, although most of the breeds have experienced complex admixture events, several breeds have preserved distinctive characteristics and can be clearly discriminated, which is likely due to the effect of different remote and/or recent genetic and demographic factors. The population structure and the low genetic diversity presented here for several breeds represent useful information to guide conservation strategies. Notably, mating plans can have an important role in restraining inbreeding and increasing the census size of these local breeds. Monitoring of inbreeding trends and improvement of the recording systems are strategic for in situ conservation of these breeds, and towards this aim, the availability of genomic data represents a fundamental resource. Moreover, these results highlighted the importance of using genomic information to reveal the genetic structure of each population and provide an objective basis for decisions regarding the conservation of the Italian local cattle breeds. When standardized genotyping arrays are adopted, it is possible to combine various datasets. Therefore, further studies are necessary to provide insights into the genetic composition and origin of Italian cattle breeds, such as the Podolian-derived breeds, using data of other worldwide cattle populations, and for the development of a SNP-based identification test for breed assignment and tracing animal products.

## Additional files

**Additional file 1.** Description of each Italian local cattle breed involved in this study [68].

**Additional file 2: Table S1.** Name of the breeds, geographic coordinates (longitude and latitude) of the center of origin, sample size before (n-PreQC) and after (n-PostQC) genotyping quality control, and origin of genotyping data.

**Additional file 3: Figure S1.** Trends in historic effective population size (Ne) (from 13 to 98 generations).

**Additional file 4: Table S2.** Pearson correlation coefficients between genetic diversity indices. Observed (Ho) and expected (He) heterozygosity, average minor allele frequency (MAF), inbreeding coefficient (*F*_HOM_), contemporary effective population size (cNe), mean ROH-based inbreeding coefficient (*F*_ROH>4Mb_) and recent and historical N_e_ estimated 13 (Ne13), 20 (Ne_20 and 80 (Ne_80) generations ago. (* p- value < 0.001).

**Additional file 5: Figure S2.** Relationship between the number of ROH and the length of the genome (Mb) covered by ROH per individual.

**Additional file 6: Figure S3.** Distribution of all ROH within breeds according to their size (kb).

**Additional file 7: Figure S4.** Model-based clustering of the estimated membership fractions of individuals from the 32 breeds analyzed in each of the K inferred clusters revealed by the ADMIXTURE software (K = 12, 16, 20, 28, 32). For a full definition of breeds see Table S1 (see Additional file 2: Table S1).

**Additional file 8: Figure S5.** Cross-validation errors of admixture analysis at different K values.

**Additional file 9: Table S3.** Pairwise *F*_ST_ values between cattle populations.

**Additional file 10: Figure S6.** Scatter plot of correlations between genetic differentiation (*F*_ST_) and geographical distances for all breeds.

**Additional file 11: Table S4.** Results of the *f3* test.

**Additional file 12: Table S5.** Results of the *f4* test.

**Additional file 13: Figure S7.** Genetic relationship defined with multidimensional scaling analysis between Italian and 62 European cattle breeds. The breeds were grouped according to their geographical origins and distributions. Northern_Europe (Belgian Blue, Dexter, Kerry, Lithuanian Light Grey, Lithuanian White Backed, Groningen Whitehead, Lakenvelder, Meuse-Rhine-Ijjsel, Norwegian Red, Finnish Ayrshire, Belted Galloway, Galloway, Angus, Scottish Highland, South Devon), England (Devon, Guernsey, Hereford, Longhorn, Lincoln Red, Milking Shorthorn, Red Poll, Beef Shorthorn, Sussex, Welsh Blach, White Park), Spain (Berrenda en Negro, Berrenda en Colorado, Cardena Andaluza, Menorquina, Pirenaica, Morucha, Mostrenca, Negra Andaluza, Toro de Lidia), France (Abondance, Aubrac, Blonde D’Aquitaine, Bretonne Black Pied, Gascon, Maine-Anjou, Maraichine, Montbeliard, Normande, French Red Pied, Salers, Tarine, Tarentaise, Vosgienne), Germany (Gelbvieh), Switzerland (Simmental, Simmentaler, Braunvieh, Ehringer), Turkey (Anatolian Black, Anatolian Southern Yellow, East Anatolian Red, South Anatolian Red, Turkish gray, Zavot). Italian breeds are colored according to their geographical distributions and are the same as those described in Fig. 2; the European breeds are colored in gray.
