# Supplementary material for: Conservation status and historical relatedness of Italian cattle breeds
Source: Genet Sel Evol. 2018 Jun 26;50:35. doi: 10.1186/s12711-018-0406-x (PMC6019226; doi:10.1186/s12711-018-0406-x)

## The Northern Italian breeds

The **PINZGAUER** is a dual-purpose (dairy and beef) local cattle breed of the alpine areas. In Europe, Pinzgauer herds are mainly located in Austria (Salzburg, Tyrol, Carinthia, Styria), Italy (South Tyrol) and Germany (Bavaria). Pinzgauer cows are very appreciated because of their hardiness, resilience and longevity, and high adaptability to highland pasture. All Pinzgauers have the typical finched pattern in common: a broad white stripe lengthwise along the whole back. The abdomen, chest, udder, and tail are white as well. Milk yield per lactation after the third calving averages 6000 kg with 4% and 3.5% of fat and protein, respectively.

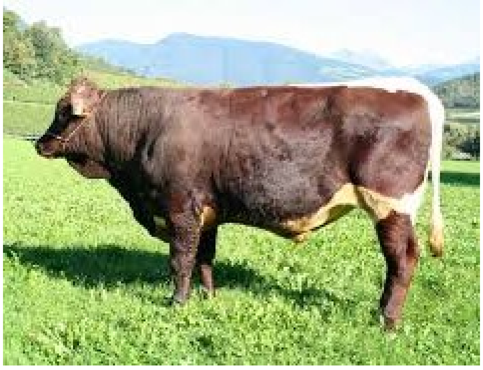

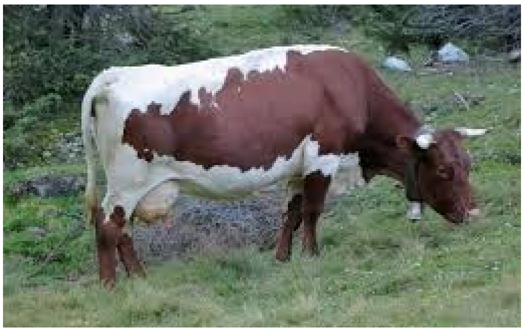

The **PUSTERTALER Sprinzen**, also called Pustertaler, is a rare cattle breed from the Puster Valley in the autonomous province of Bolzano. The Pustertaler was highly esteemed for its milk, and commanded high prices. Cattle are of medium-large size. Head, rib and distal parts of the limbs are spotted (black or red-brown, in various shades); the dorsal-lumbar line, the abdomen and the perineum are white. The horns, present in both sexes, are heavy, medium-long, white with dark apices, directed laterally, up and forward. Pustertaler cattle are robust and tough animals. They are characterized by longevity and easy calving. Calves are very vital, with high daily weight increase when feeding on basic rations. Additionally, meat quality is very good.

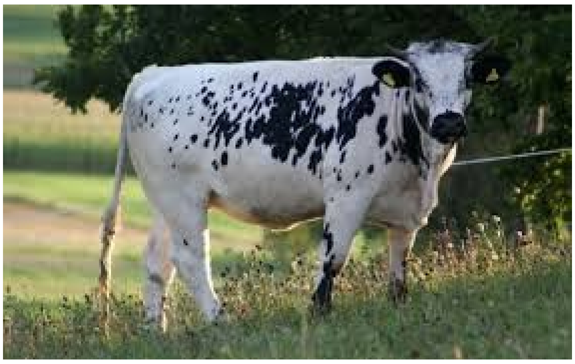

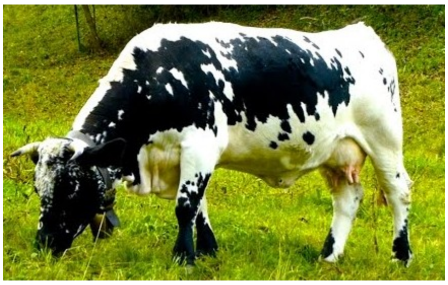

The **BARÁ-PUSTERTALER** breed derives from the crossbreeding of Pinzgau breed cattle with other red pied or black pied cattle reared since XVIII century in the mountain region of Val Pusteria (Alto Adige, NE Italian Alps). The Barà breed of the province of Turin is closely similar to the Pustertaler; the two populations have been treated and registered as a single breed. Today there are about 5,000 heads, mainly reared in the provinces of Turin. It is a breed with a balanced dual purpose attitude (milk and meat). The milk is used for the production of typical cheeses, such as Toma and Cevrin di Coazze (mixed cow and goat milk cheese from Val Sangone). The meat obtained from medium-weight beef, is characterized by a good infiltration of marbling fat. It is a breed very suitable for the alpine environment, able to graze even in harsh environments. The Barà-Pustertaler is also reared according to the cow-calf line (a form of breeding that provides for the stay of the calf next to the mother for the period of lactation).

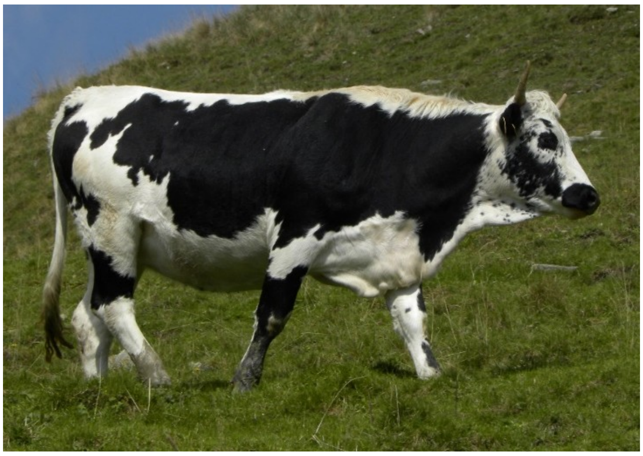

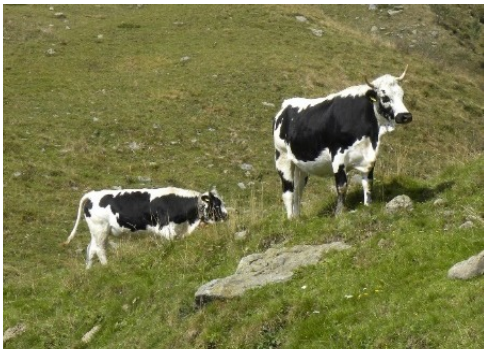

The **RENDENA** is a local dual purpose breed native of the homonymous valley in Trento province and widespread in the north-east of Italy. The coat of Rendena is characterized by different shades of dark brown, almost black in males, with a lighter dorsal stripe and a white ring around the black muzzle. Rendena cattle are characterized by small to medium body size and account for 3,985 officially registered cows reared in about 200 farms. The breeding goal of Rendena cattle is to improve both quality and quantity of milk not losing the meat attitude. Typical products obtained from RENDENA are cheeses, mainly in the mountain area of origin like Spressa and meat from bulls commercialized in agro-touristic contests in Veneto region.

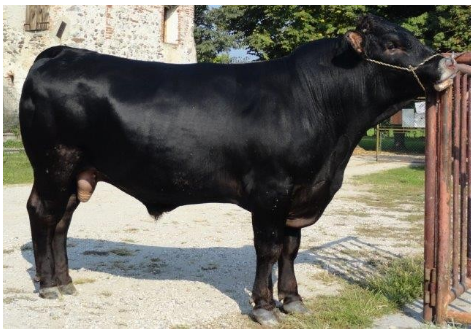

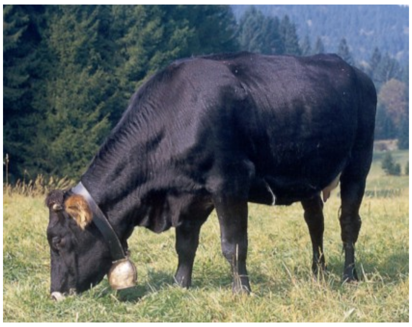

The **BURLINA** is a native, Italian, dairy cattle breed reared in North-East Italy; it is a small sized animal with black spotted coat, well adapted to difficult environmental conditions as marginal mountain areas; thanks to its good grazing characteristics. The origin of Burlina is still uncertain. The number of reared animals drastically decreased from 15 000 in 1930 to 2300 in 1972. Nowadays, about 350 cows are registered in the Italian Herd Book, most of them located in the Treviso and Vicenza provinces of North-East Italy. Several actions have been developed to safeguard the Burlina breed, especially in the 1980s, aiming to increase the number of reared animals and the within population variability and to minimize the crosses with other breeds. Traditionally, from Burlina milk a typical cheese called Morlacco has been produced; it is a raw whole milk cheese preferably obtained from cows at pasture to achieve a typical herbs flavour

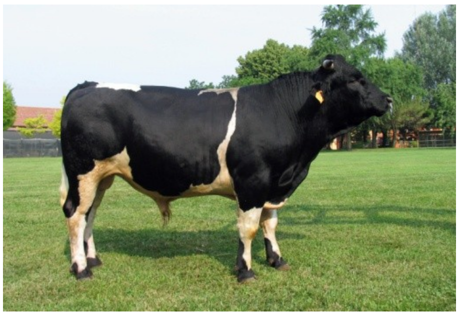

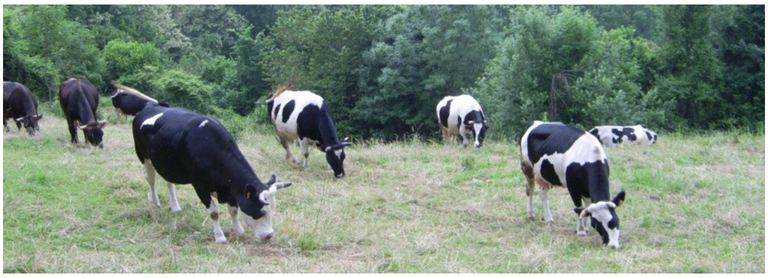

The **VARZESE-OTTONESE** is a local population raised mainly in the Lombardy region (Northern Italy). The Varzese-Ottonese is small sized dual-purpose breed with a pheomelanic and uniform coat colour (dark golden red). Currently the breed register counts 295 animals of which 60 males, and is defined as “breed at risk” by FAO. Rustic and long lived, mainly suitable to labour with modest quality milk and meat. The local breeder association realized many actions of conservation and valorization of its dairy products as Montebore, Menconico and Nisso cheese.

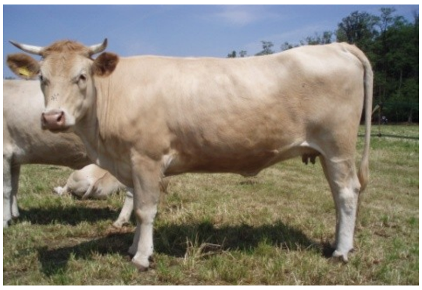

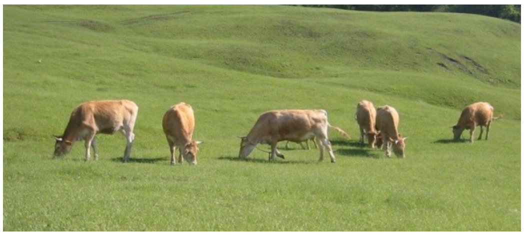

The **PEZZATA ROSSA D’OROPA** breed is native from Valle Elvo (Biella, NW Italian Alps). The breed is nowadays bred in Piedmont, and a has been included by the FAO in the world list of endangered breeds. There was a recent recovery of the population size, which currently has about 5,000 heads. The PRO is a medium-sized cattle. The coat is red-spotted (the black spotted genotype is not frequent), while the head, limbs, abdomen and the tail are white. The eyes are frequently

surrounded by round red spots; the muffle is pink. The horns are of medium length, yellowish and worn up and forward. It is a dual-purpose breed (milk and meat), with a predominance for milk production. The milk can be used for the production of typical butter and cheeses, such as Toma Biellese, Maccagno, Tuma brüsca, Bëddu and Tomino di Sordevolo. The traditional breeding system provides for the housing in the winter months and the practice of the summer mountain pasture. The breed, excellent grazing cow, is well adapted to the mountain environment and has always been appreciated for rusticity and frugality.

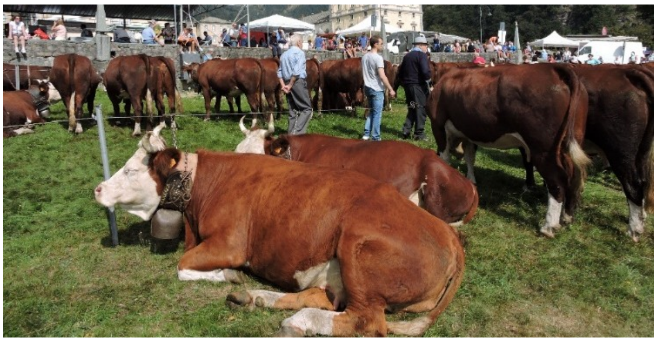

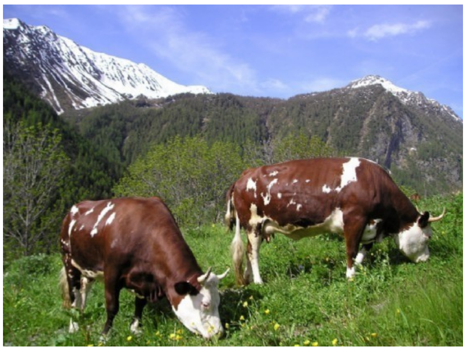

The **CABANNINA** is an autochthonous breed with dual purpose, native of the Cabanne plain, in the higher part of Aveto Valley in the province of Genova. It is bred in marginal condition not suitable for cosmopolitan breeds. The Cabannina is characterized by medium size animals with an eumelanic brown and uniform coat colour, often with a lighter mule stripe on the back. The breed standard dates 1982, since 1992 FAO classified this breed at risk. Currently the breed register, opened in 1985, counts 241 animals of which 37 males (AIA, 2015), thanks to the effort of the local breeder association realized also through the valorization of its dairy products as the “U Cabanin” cheese made exclusively with Cabannina milk.

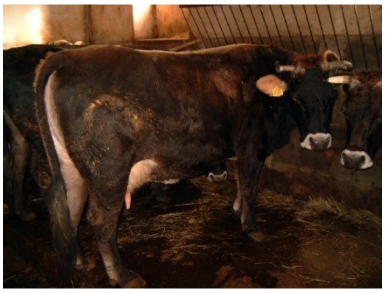

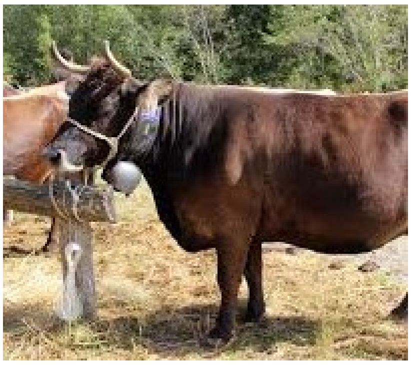

The **PIEDMONTESE** cattle is a beef breed of medium size that converts forage very well into beef and has a high dressing out percentage. The quantity of commercial cuts is higher than that of bigger-sized breeds. It has very fine bones, a fine and elastic skin, a low quantity of external fat and lean and tender, but tasty meat. The bulls have a grey or pale fawn coat, with black hairs on the head (especially around the eye sockets), on the neck, the shoulders, the distal regions of the limbs and sometimes on the lateral faces of the body and the hind limbs. The cows have a white or pale fawn coat with shades of grey or pale fawn. At birth the coat of the calves is of a deep pale fawn colour. Despite their size, the weight gain of Piemontese cattle is high, reaching 1.4 kg a day in the calves between weaning and slaughtering, when feeding and environmental conditions are optimal. The milk production of the Piemontese is sufficient to suckle the calf. Some of the Piemontese breeders use the milk for cheese production. The cheeses "Castelmagno", "Bra", "Raschera" and many of the "Tome" coming from the valleys of the region Piemonte are produced with Piemontese milk. The particular characteristic of the Piemontese cattle breed is the muscular hypertrophy, better known as the 'double muscle factor'.

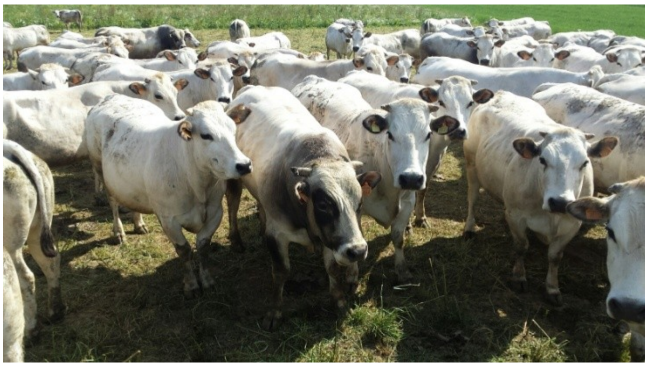

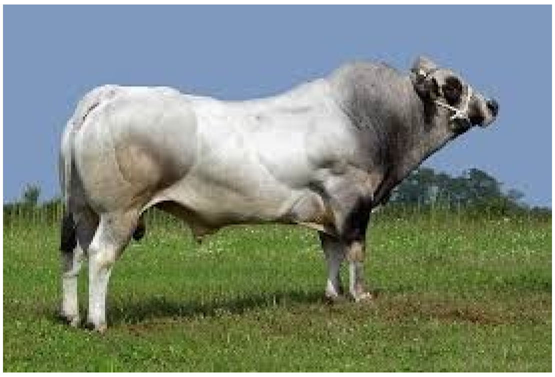

**Northern-central Italian**

The **REGGIANA** is a local dairy cattle breed reared mainly in the North of Italy, in the province of Reggio Emilia. The population of cows of this breed was about 40,000 animals in the 1940s; this number decreased progressively till the 1980s when it reached about 500 cows; subsequently it increased, reaching the current number of about 2000 cows. The breed is characterized by the red wheat colour coat. The animals of this breed have a medium size. The breed preserves excellent reproductive, rusticity performances and a good quality of meat produced by the fatted cows. Mean milk yield production of the cows of this breed is about 30% less than that of Holstein cows. To overcome production limits of this breed, a new brand of Parmigiano Reggiano cheese made only of Reggiana milk has been developed.

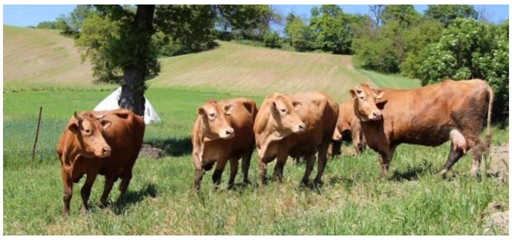

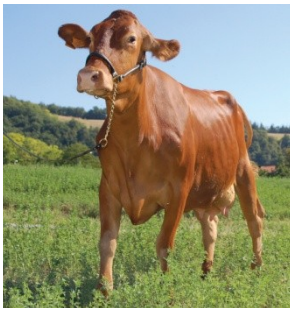

The **MODENESE** is a breed of dual-purpose cattle from the Po Valley, in the Emilia Romagna and Lombardy regions of northern Italy. It is reared for beef and milk production, but in the past was a triple-purpose breed, used also as a draught animal. The name derives from that of the province of Modena, where it is thought to have originated. Today approximately 650 cows are reared. The breed shows a white mantle shading to grey at the shoulder and thigh levels, yellowish medium-sized horns with black tip; the frontal hair tuft may have some reddish shading as it comes from dark golden cattle. Depigmented skin, medium-small size and height. Milk is sold to local market or industry, linked to the production of Parmigiano Reggiano cheese, anonymous and Modenese branded.

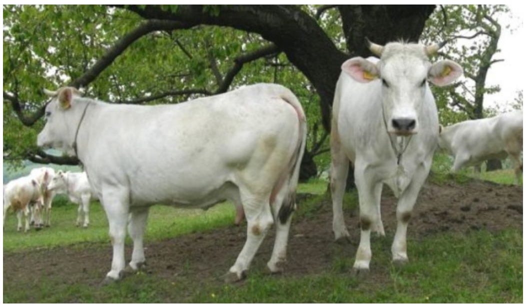

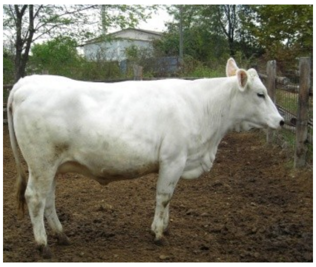

The **PONTREMOLESE** is a cattle breed from Tuscany in central Italy. In the past it was used for the production of oxen that were requested on the market by Ligurian and Lombard farms, but above all the local ones for the transport of marble from the quarries of the Apuan Alps to the sea. It is a medium sized breed. The bull has a dark golden red coat with a long light stripe along the dorsal backbone with darker shades on the head (with eyeshadows) on either sides on the neck, on the external part of the shoulders, on the frontal sides of the forearms and of the cannon-bones, on the coronary pad of the four limbs and on the lower part of the sides of the trunk. The wide muzzle is dark black; the head is quite light with a straight profile. The trunk is quite short with raised withers as compared to the dorsal backbone. As to the cows, the coat is light golden red with slightly darker shades in the above mentioned parts in the bull. The head is a bit lighter and longer than the bull's. Very rustic breed with quite good milk production. Currently only breeding for meat production could be pursued.

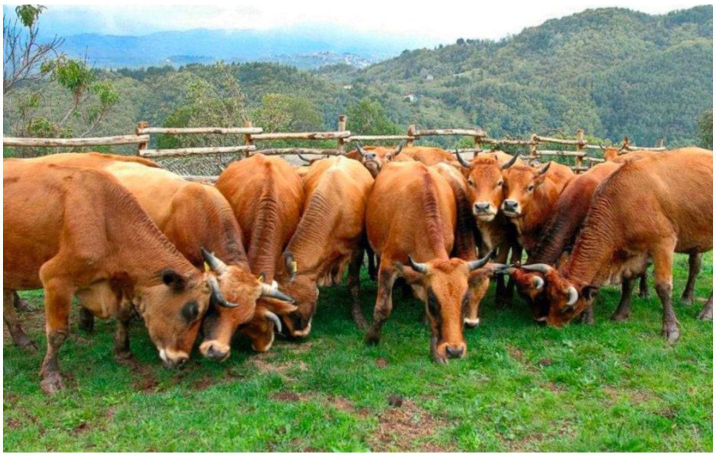

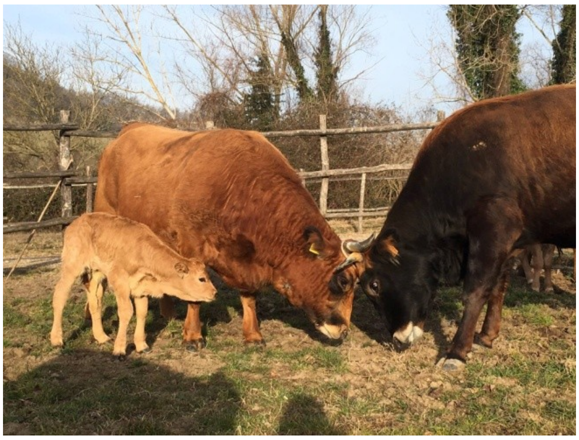

The **GARFAGNINA** is a local beef cattle native of Tuscany. The breed originated from Garfagnana valley (Lucca province) and it is also reared in Lunigiana valley as well as in the surrounding of Lucca. Currently, only 55 individuals are enrolled in the herd book on a total of 162 animals identified and it is considered "at risk" by FAO. It is a medium size breed characterized by a coat variable from light to dark grey. The breed was recognized for its good ability to use rough forages as well as wood products (chestnuts, green leaves, …) and it is well adapted to the conditions climatic of the breeding area, sometimes severe. It is currently considered a double purpose breed with milk production as main aptitude. Today the Garfagnina breed is raised with a semi-extensive management with the animal maintained in the pasture during all the summer period without receiving any kind of nutritional supplementation.

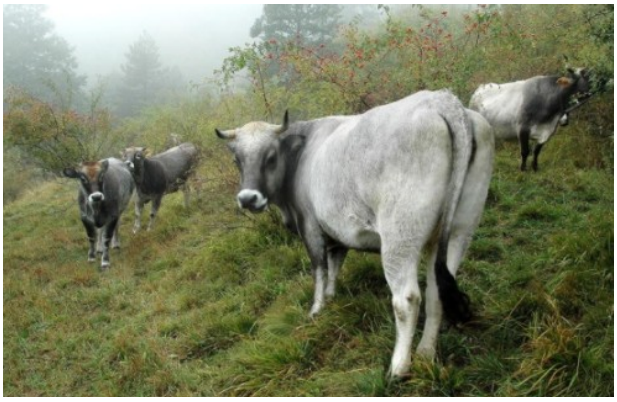

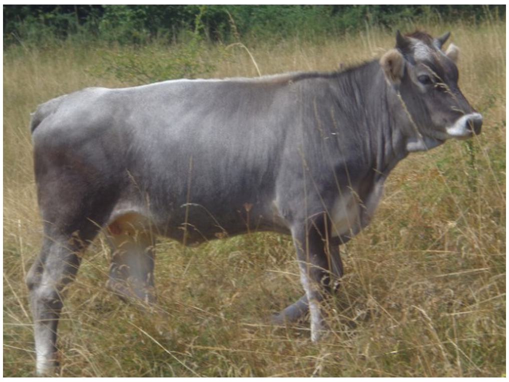

The **MUCCA PISANA** is a breed reared around Pisa's province (Italy). Its origin is very uncertain, because numerous hypothesis were proposed, but it is surely that it derived by a different no-autochthonous breeds. First news on this breed was proposed between the XVIII and XIX centuries, which revealed the introduction of Schwyz breed in Pisa's zone to improve meat production. The most accepted theory was the crossbreed of a local breed with Brown Swiss. Despite these different information, it is sure that Mucca Pisana was the results of intense work of breeding program using Swiss breeds. Moreover, other breeds contributed to define Mucca Pisana: for several years Brown Swiss was most used for

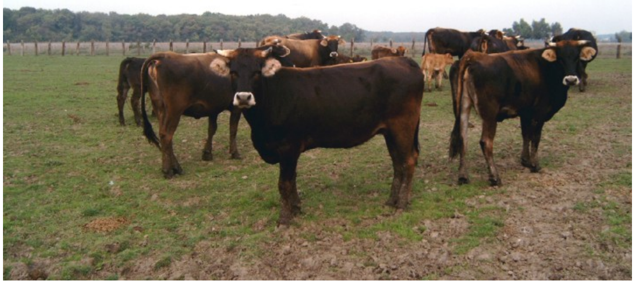

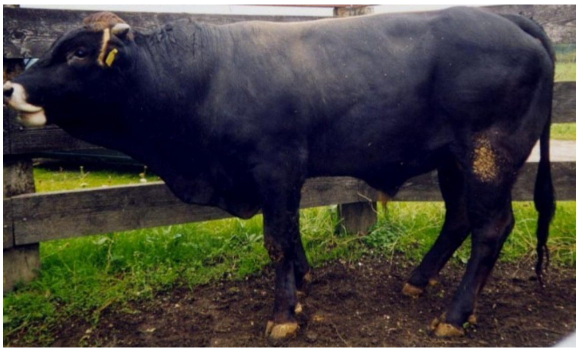

crossbreed, while from 1850 were known the use of Shortons and Charolais. Surely, the most longer crossbreed was carried out with Chianina, which increased the size and resistance to work. These characteristics are well evident in the actual population of Mucca Pisana.

**Podolian-derived breeds**

The **CALVANA** is a local beef cattle originated from Calvana mountain, a bald and stony area north of Prato province. The breed is then expanded in other areas of Tuscany hills. Currently, 382 individuals are enrolled in the herd book and the breed is considered "at risk" by FAO. The breed is considered originated to prolonged cross-breeding of existing animals raised in the region (Podolica blood) with the Chianina cattle breed. It

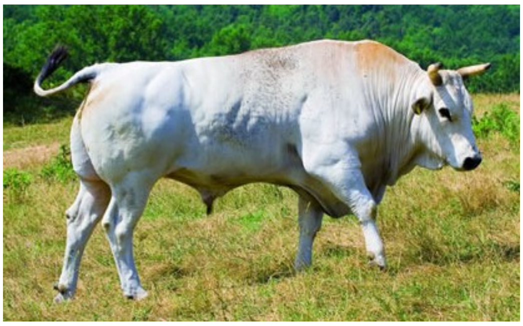

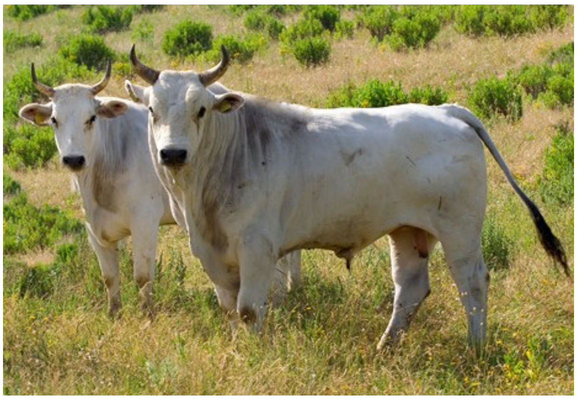

is a medium-large breed in size, with pigmented skin and porcelain white coat, even if during the first months of life the coat is golden-blonde. Currently it is a beef breed with good grazing ability and able to well exploit the marginal environments and the rough pastures of the Apennines. Meat of Calvana cattle is used to produce the typical 'Florentine steak 'dish.

The **CHIANINA** originates in the area of the Valdichiana, from which it takes its name. Chianina oxen were the principal source of agricultural power in the area until displaced by mechanization. From 1931 breeders began to favour selection of animals more suited to meat production, with shorter limbs, longer bodies and more heavily muscled rump and thighs; recently, selection is based also on factors such as growth rate, meat yield and, in cows, maternal ability.

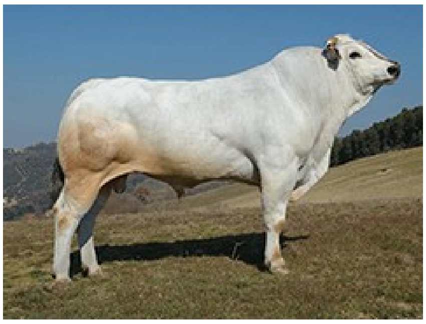

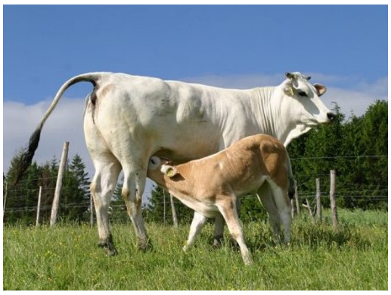

Through exportation of breeding stock, of frozen semen and of embryos, it has reached China, Russia, Asian countries and the Americas. The Chianina is both the tallest and the heaviest breed of cattle. The coat of the Chianina is white; very slight grey shading round the eyes and on the foreparts is tolerated. The Chianina is one the most important meat breed.; the milk is barely sufficient for suckling. In beef production, Chianina cattle are chosen for their growth rate, which may exceed 2 kg per day, the high yield and high quality of the meat, and their tolerance of heat and sunlight. They have better resistance to disease and insects than many other domestic cattle.

The **ROMAGNOLA** is a cattle breed from the Emilia-Romagna region. Romagnola cattle were used principally as draught beasts in the past. Romagnola cattle are ivory-white, tending to grey on the foreparts, particularly in bulls; the skin and natural openings are black. The colour of the coat varies with the season, and is darker in winter. The horns are light, lyre-shaped in cows, half-moon-shaped in bulls; they are slate-grey in young animals, becoming pale at the base and dark at the tip with maturity. As with all Podolic cattle, the calves are born wheat-coloured but become white at about three months. Its adaptability to difficult environments and its dislike of stall housing make it an excellent grazing animal. The breed is used for its meat, and the traditional farming method, involving free grazing during the summer months and an exclusively natural diet, guarantees beef of superior quality. The best cuts, especially the large, flavorful chops, stand up well to comparison with the more famous Chianina. Roast rump or tenderloin, cooked slowly and gently according to Romagna tradition, are also excellent.

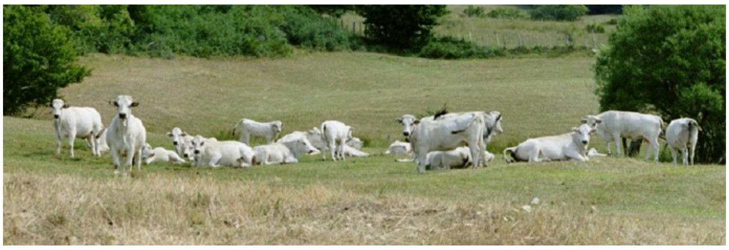

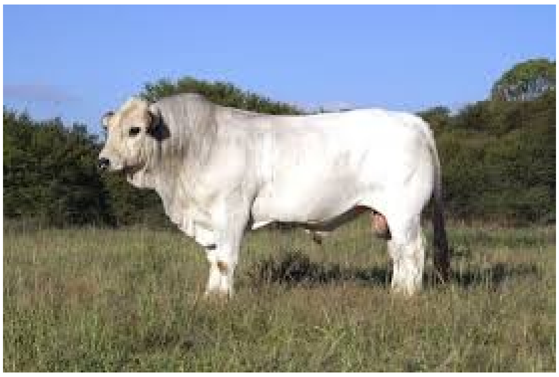

The **MARCHIGIANA** can be defined as a "synthetic" beef cattle breed created in Italy. The breed was developed by crossing native Podolian cattle with the Chianina and Romagnola breeds in the late 19th and early 20th century. The breed was specifically originated in the Marche region. Although the breed is not as tall as the Chianina, but today it still bears a close conformational resemblance to the Chianina cattle. It is a short haired animal and it's color vary from light grey to almost white. Their muzzle, tongue and external opening are black and their skin is pigmented. They are generally dark around the eyes and their tail switch is dark. They can be either horned or polled. The Marchigiana cattle are raised primarily for meat production. They were also used for draft work as oxen prior to the 1950s. Marchigiana cattle are muscular with a fine bone structure. They grow relatively faster and have a very high feed conversion ratio. They have great disease resistance power and can adapt themselves easily to almost any climatic conditions.

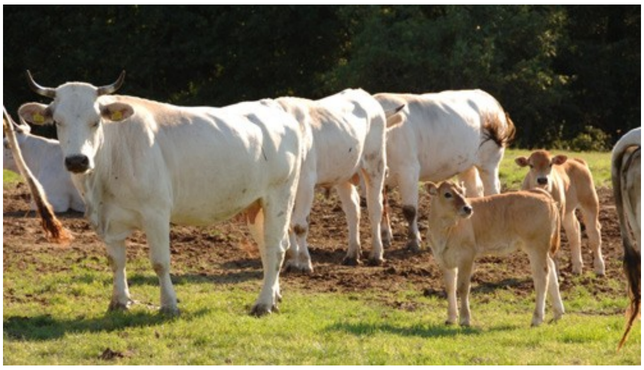

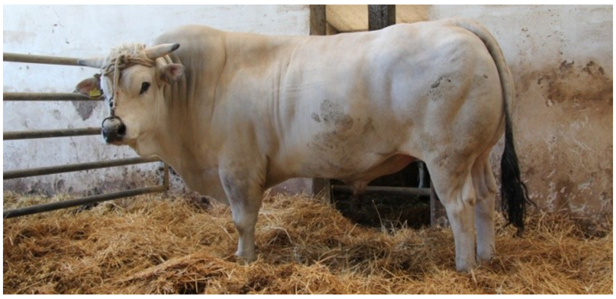

The **MAREMMANA** is reared in the Maremma, a former marshland region in southern Tuscany and northern Lazio. For centuries large herds of Maremmana cattle were raised in the malarial marshlands of the Maremma, herded by the *"Butteri"*. Following the drainage of the marshes the consequent destruction of the wetland habitat, efforts were made to improve the breed, and in particular to increase its body weight, with considerable successThe Maremmana is grey; males are darker than females, especially on the foreparts. The muzzle, hooves, switch and lower part of the scrotum are black; the skin is black, but some depigmentation at natural openings is tolerated. As in other breeds of Podolic origin, calves are born wheat-coloured and become grey at about three months old. The horns are robust; in males they are of half-moon shape, in females lyre-shaped. They are slate-grey in young animals, and become whitish with black tips in adulthood. The Maremmana was formerly used as a draught animal, principally in agriculture and forestry, but also for haulage work, for example in the marble quarries of Monte Amiata. It is one of the two breeds used in the preparation of the "bistecca alla fiorentina".

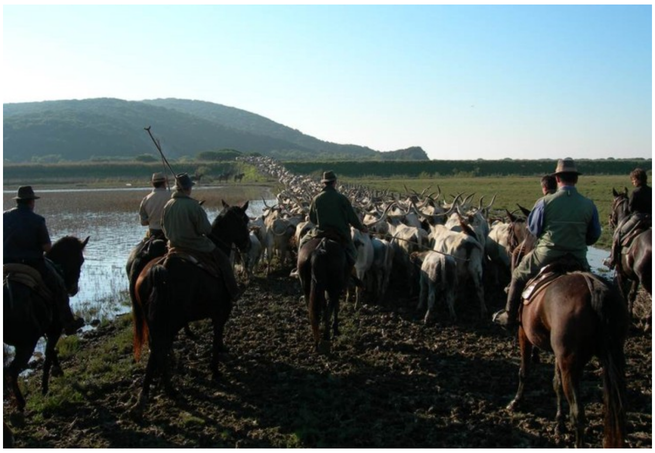

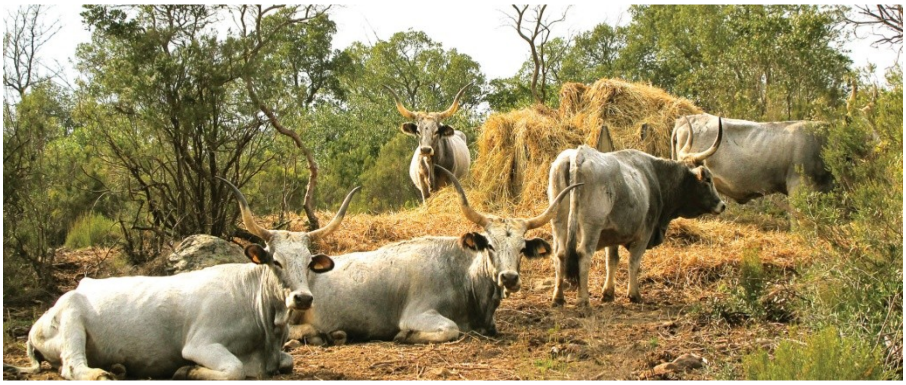

The **PODOLICA** is one of the oldest breed of Italy, and is characterized by grey coats and upright horns. The calf has a typically fawn colour coat. The breed is particularly adapted to the harsh environment of the hilly and mountainous area of Central and South Italy. The breed was originally triple purpose but recently a selection scheme aimed to improve the meat production has been implemented. Nevertheless

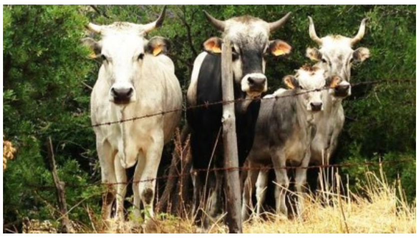

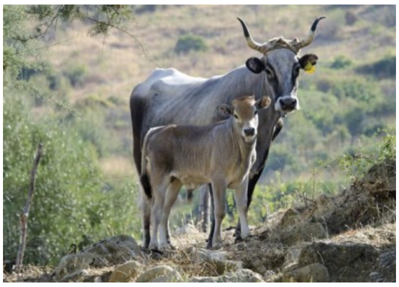

the milk production is not negligible and a renowned cheese (Caciocavallo Podolico) is produced. At the present Podolica is reared in the regions of Southern Italy and consists of more than 30.000 heads recorded in the herd book.

**The southern, Sicilian and Sardinian breeds**

The **AGEROLESE** is a local Italian cattle breed reared in the province of Naples, in the proximity of the town of Agerola. It is a dual-purpose breed, which originated during the nineteenth century from an autochthonous nucleus of Podolian cows crossed with Swiss Brown, Dutch Friesian, and Jersey bulls. The population is considered endangered by the FAO. The colour of the coat can vary from brown to black with a ridge of light hairs around the muzzle, which is dark as well, medium sized light-coloured horns, bent sideway and forward. Meat and milk production are of high quality, although they can vary according to the difficulties of the breed to adapt to difficult or marginal surroundings. The milk of this cow is used mainly for the production of 'Provolone del Monaco'.

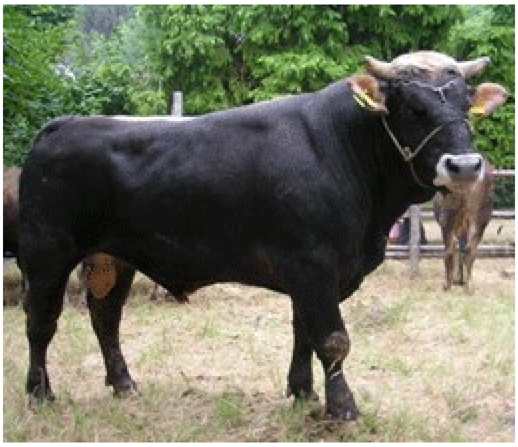

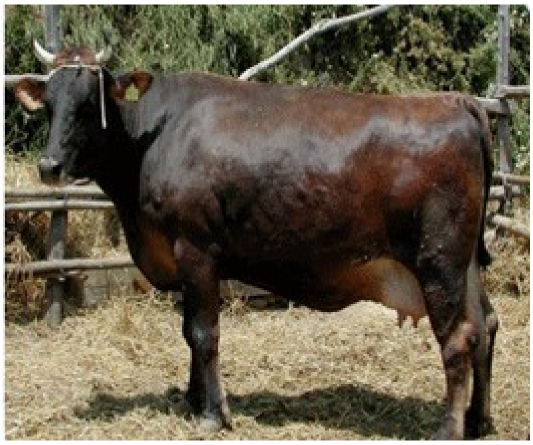

The **CINISARA** is a local dairy cattle belonging to the group of the Podolian breeds. The breed is native of the town of Cinisi (hence its name) in the province of Palermo and it is reared in a restricted area of Sicily (northwest). Currently, about 5,000 animals are enrolled in the herd book. It is a medium sized animal characterized by a uniform black coat and, less frequently, speckled with spots called ‘agghi’ in the local dialect (black with a white band on the head, dorsal line, perineum, tail and ventral line). This breed, thanks to the natural adaptation to harsh environment and climates, is able to use poor quality feeds and to cope with endemic diseases. The milk is used to produce the Caciocavallo Palermitano, a “pasta filata cheese”.

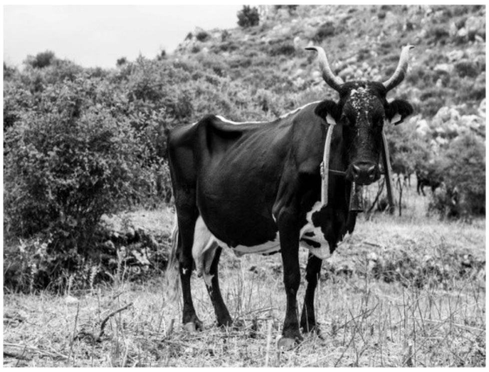

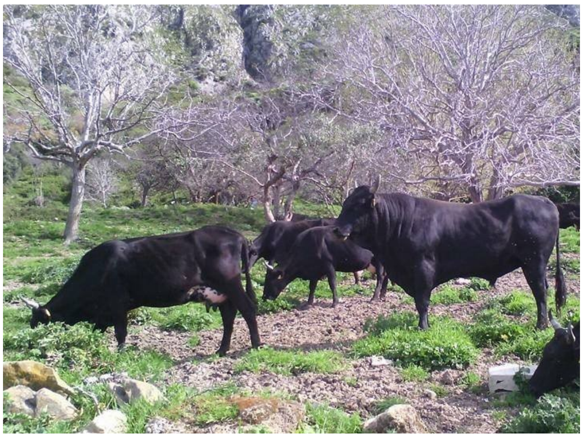

The **MODICANA** is native of the ex-county of Modica (province of Ragusa-Sicily). Very rustic and frugal breed, lately it has spread around the whole region being easily apt to any pedoclimatic situation. It is characterized by a uniform dark red coat shading from the black of the bull to the golden red of the cow. Black shades particularly in the front or external sides of the thighs and black tail tuft. The male has a darker coat, dark red wide muzzle with dark black nostrils. Black hoofs. Yellowish horns at the basis and black at the tips. Large udders with long big teats. Moderate size and height, a very sharp figure but very solid skeleton. Very good working characteristics, quite good for milk production (considering the extremely poor diet) and quite poor for meat. It used to be bred for labour (limbs and hoofs are very strong), today it is bred for milk, with which some typical cheeses are made (Caciocavallo and Ragusano).

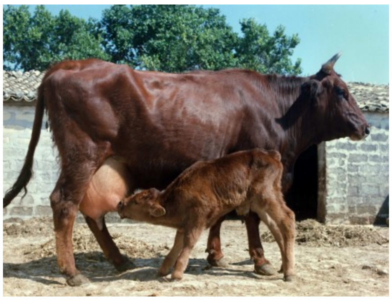

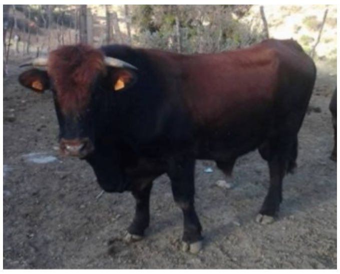

The **ROSSA SICILIANA** is a red cattle population exclusively reared in Sicily. Today this cow is considered a subpopulation of Modicana breed. It has a coat color ranging from the blond to the dark red with shade accentuations towards vinous and black. It is mainly reared in the mountain range of the Peloritani, Nebrodi and Madonie in Sicily; thanks to its particular agility, it exploits difficult territories in disadvantaged pedo-climatic environments. The breed is among the morphotypes with a tendentially long-line constitution, therefore with an attitude prevalent to milk production; particularly, during the period of higher forage availability, its milk is used for the production of the "Provola dei Nebrodi".

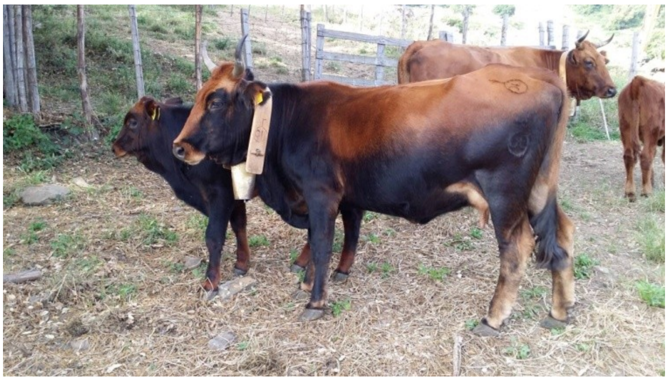

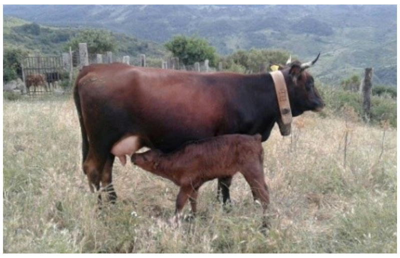

The **SARDA** breed is an autochthonous breed from the Sardinia island. It is a low sized animal, with high variability of color coat with presence, sometimes, of streaks of various colors and amplitude. It is mainly reared around the main mountainous areas of the island.

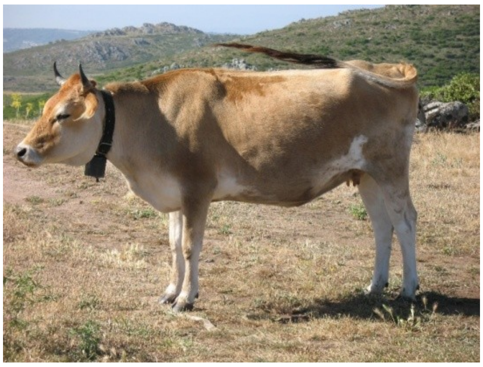

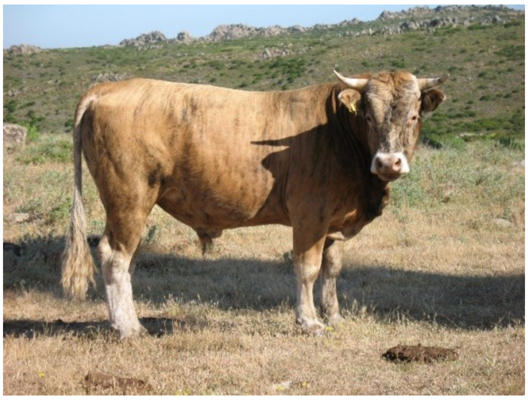

Currently, the breed counts around 25.000 heads. Thanks to its natural adaptation to harsh environment, its calving ease and the good maternal aptitude, the breed is mainly exploited as maternal breed for terminal crosses with specialized meat breeds.

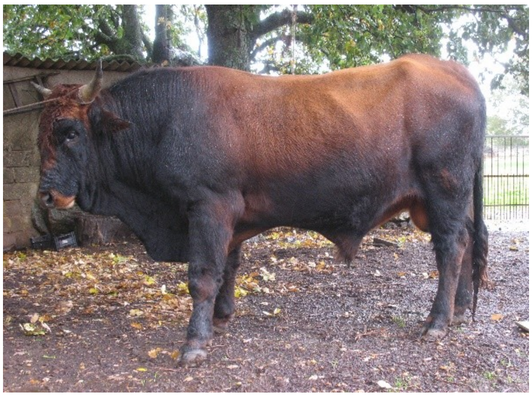

The **SARDO-MODICANA** breed originated from the upgrading of local cattle population by crossing with bulls from the Modicana breed imported in Sardinia since the end of the XIX century. It is a medium size breed (average live weight around 650 and 420 kg for adult males and females, respectively). Coat color varies from light red almost uniform in females to red vinous, darker in the neck and head, in males. At present, it is mainly reared in the western region of Sardinian called “Montiferru”. Around 3,000 animals are registered in the herd book. Originally exploited also for labor, the breed is nowadays used for milk and meat production. The milk is mainly transformed into a traditional cheese named “Casizolu”.

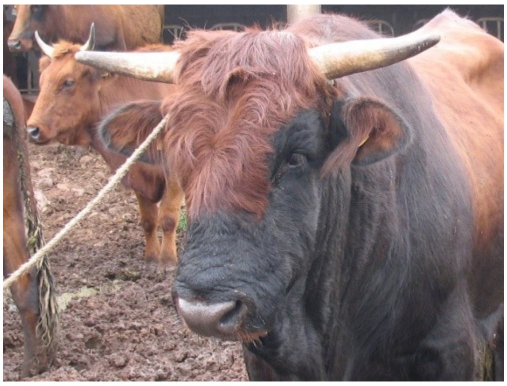

Supplement: Supplementary file 1 — Additional file 1. Description of each Italian local cattle breed involved in this study [68]. [file 12711_2018_406_MOESM1_ESM.pdf]
