# Supplementary material for: Conservation status and historical relatedness of Italian cattle breeds
Source: Genet Sel Evol. 2018 Jun 26;50:35. doi: 10.1186/s12711-018-0406-x (PMC6019226; doi:10.1186/s12711-018-0406-x)
Supplement: Supplementary file 2 — Additional file 2: Table S1. Name of the breeds, geographic coordinates (longitude and latitude) of the center of origin, sample size before (n-PreQC) and after (n-PostQC) genotyping quality control, and origin of genotyping data. [file 12711_2018_406_MOESM2_ESM.docx]

**Additional File 2: Table S1** Name of the breeds, geographic coordinates (longitude and latitude) of the center of origin, sample size before (PreQC) and after (PostQC) genotyping quality control, and origin of genotyping data.

| **Breed name** | **Breed code** | **Longitude** | **Latitude** | **PreQC** | **PostQC** | **Data origin** |
| --- | --- | --- | --- | --- | --- | --- |
| AGEROLESE | AGER | 14,54 | 40,64 | 24 | 22 | This study |
| BARA’-PUSTERTALER | B-PUS | 7,118 | 44,8095 | 24 | 24 | This study |
| BURLINA | BURL | 11,84 | 45,83 | 24 | 24 | This study |
| CABANNINA | CABN | 9,35 | 44,55 | 24 | 22 | This study |
| CALVANA | CALV | 11,15 | 43,81 | 24 | 24 | This study |
| CHAROLAIS | CHAR | 4,28 | 46,43 | 25 | 25 | Decker et al., [14] |
| CHIANINA | CHIA | 11,73 | 43,41 | 23 | 23 | This study |
| CINISARA | CINI | 13,11 | 38,15 | 30 | 30 | Mastrangelo et al., [16] |
| GARFAGNINA | GARF | 10,41 | 44,15 | 24 | 23 | This study |
| ITALIAN BROWN | IT-BR | 8,23 | 46,81 | 32 | 32 | This study |
| ITALIAN HOLSTEIN | IT-HO | 5,29 | 52,13 | 32 | 32 | This study |
| ITALIAN SIMMENTAL | IT-SI | 12,12 | 45,70 | 32 | 32 | This study |
| LIMOUSIN | LIMO | 1,26 | 45,83 | 20 | 20 | Matukumalli et al., [13] |
| MARCHIGIANA | MARC | 13,45 | 43,30 | 22 | 22 | This study |
| MAREMMANA | MARE | 11,11 | 42,76 | 25 | 24 | This study |
| MODENESE | MODE | 10,93 | 44,65 | 24 | 23 | This study |
| MODICANA | MODI | 14,76 | 36,86 | 30 | 29 | Mastrangelo et al., [16] |
| MUCCA PISANA | M-PIS | 10,35 | 43,68 | 24 | 23 | This study |
| PEZZATA ROSSA D’OROPA | PRDO | 12,20 | 46,25 | 24 | 23 | This study |
| PIEDMONTESE | PIED | 7,7 | 45,01 | 21 | 21 | Decker et al., [14] |
| PINZGAU | PINZ | 12,33 | 46,75 | 24 | 24 | This study |
| PODOLICA | PODO | 15,67 | 40,95 | 24 | 24 | This study |
| PONTREMOLESE | PONT | 10,41 | 44,11 | 24 | 24 | This study |
| PUSTERTALER | PUST | 11,35 | 46,50 | 24 | 24 | This study |
| REGGIANA | REGG | 10,63 | 44,70 | 30 | 26 | Mastrangelo et al., [46] |
| RENDENA | REND | 10,76 | 46,13 | 24 | 24 | This study |
| ROMAGNOLA | ROMG | 12,04 | 42,22 | 21 | 21 | Matukumalli et al., [13] |
| ROSSA SICILIANA | R-SIC | 14,44 | 38,02 | 24 | 24 | This study |
| SARDA | SAR | 9,17 | 40,45 | 30 | 30 | This study |
| SARDO-BRUNA | S-BRU | 8,55 | 40,72 | 10 | 10 | This study |
| SARDO-MODICANA | S-MOD | 8,59 | 40,22 | 28 | 28 | This study |
| VARZESE-OTTONESE | VR-OT | 9,19 | 45,46 | 43 | 43 | This study |
