# Supplementary figures and images for: Conservation status and historical relatedness of Italian cattle breeds
Source: Genet Sel Evol. 2018 Jun 26;50:35. doi: 10.1186/s12711-018-0406-x (PMC6019226; doi:10.1186/s12711-018-0406-x)

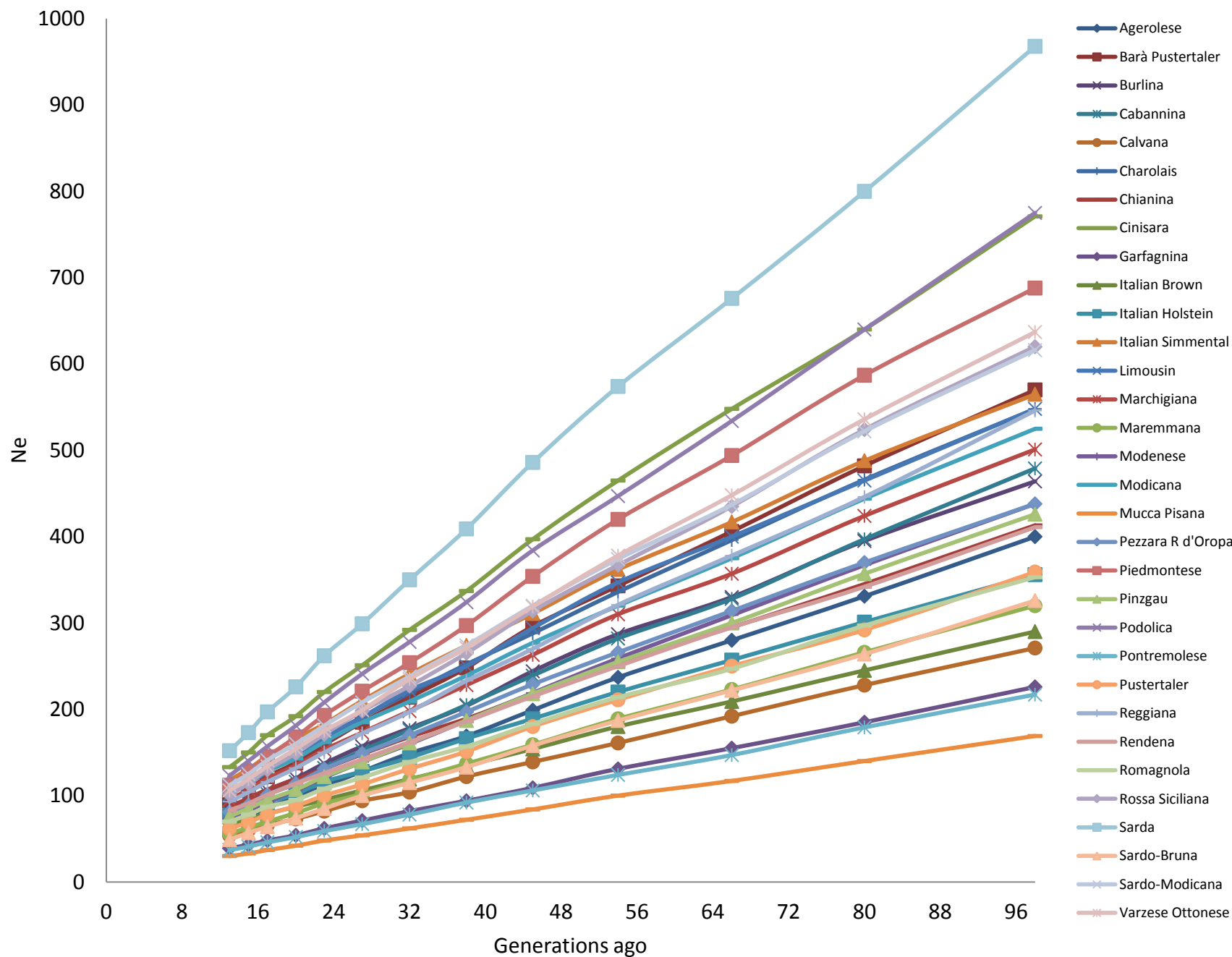

Supplement: Supplementary file 3 — Additional file 3: Figure S1. Trends in historic effective population size (Ne) (from 13 to 98 generations). [file 12711_2018_406_MOESM3_ESM.pdf]

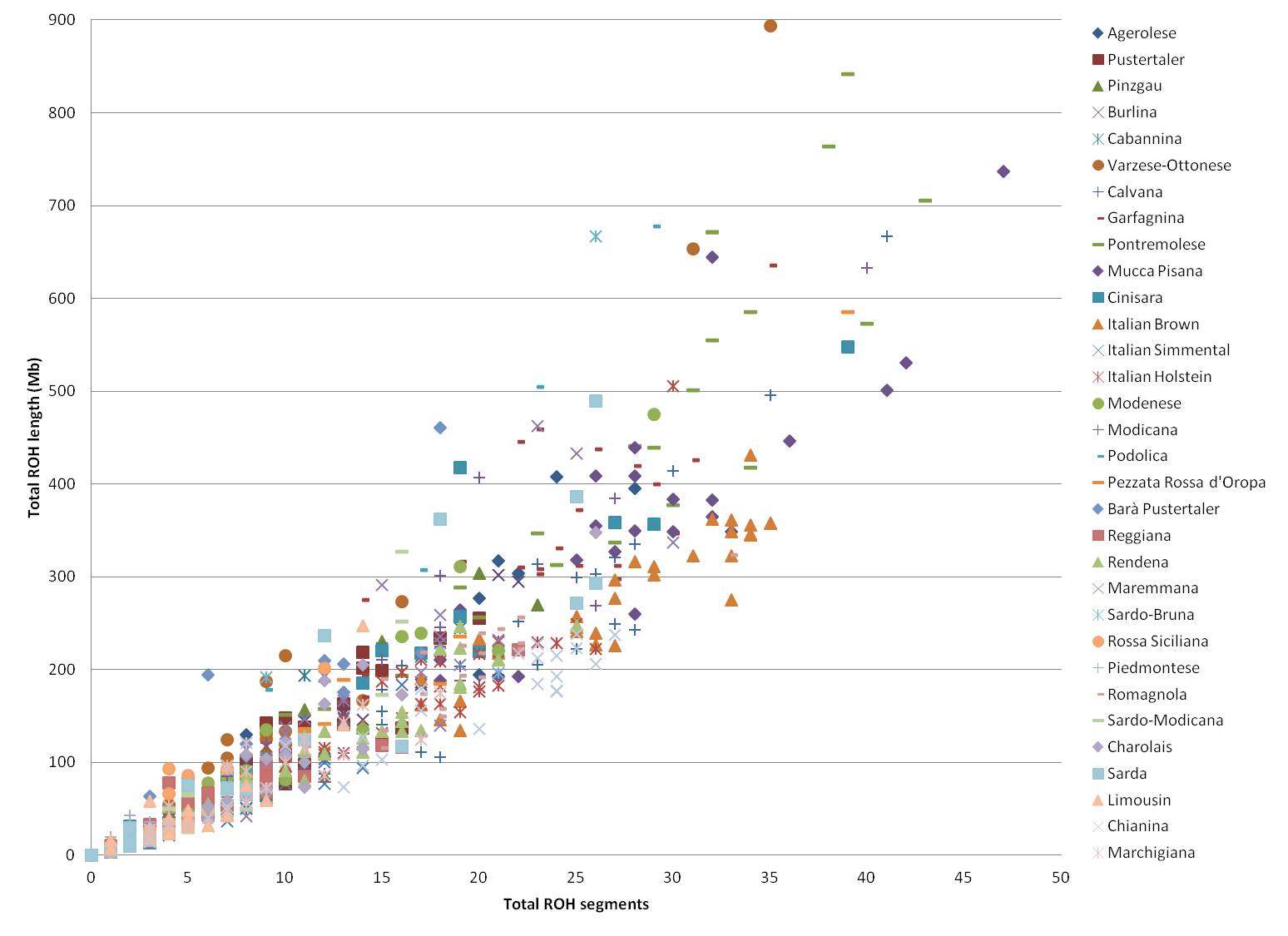

Supplement: Supplementary file 5 — Additional file 5: Figure S2. Relationship between the number of ROH and the length of the genome (Mb) covered by ROH per individual. [file 12711_2018_406_MOESM5_ESM.jpg]

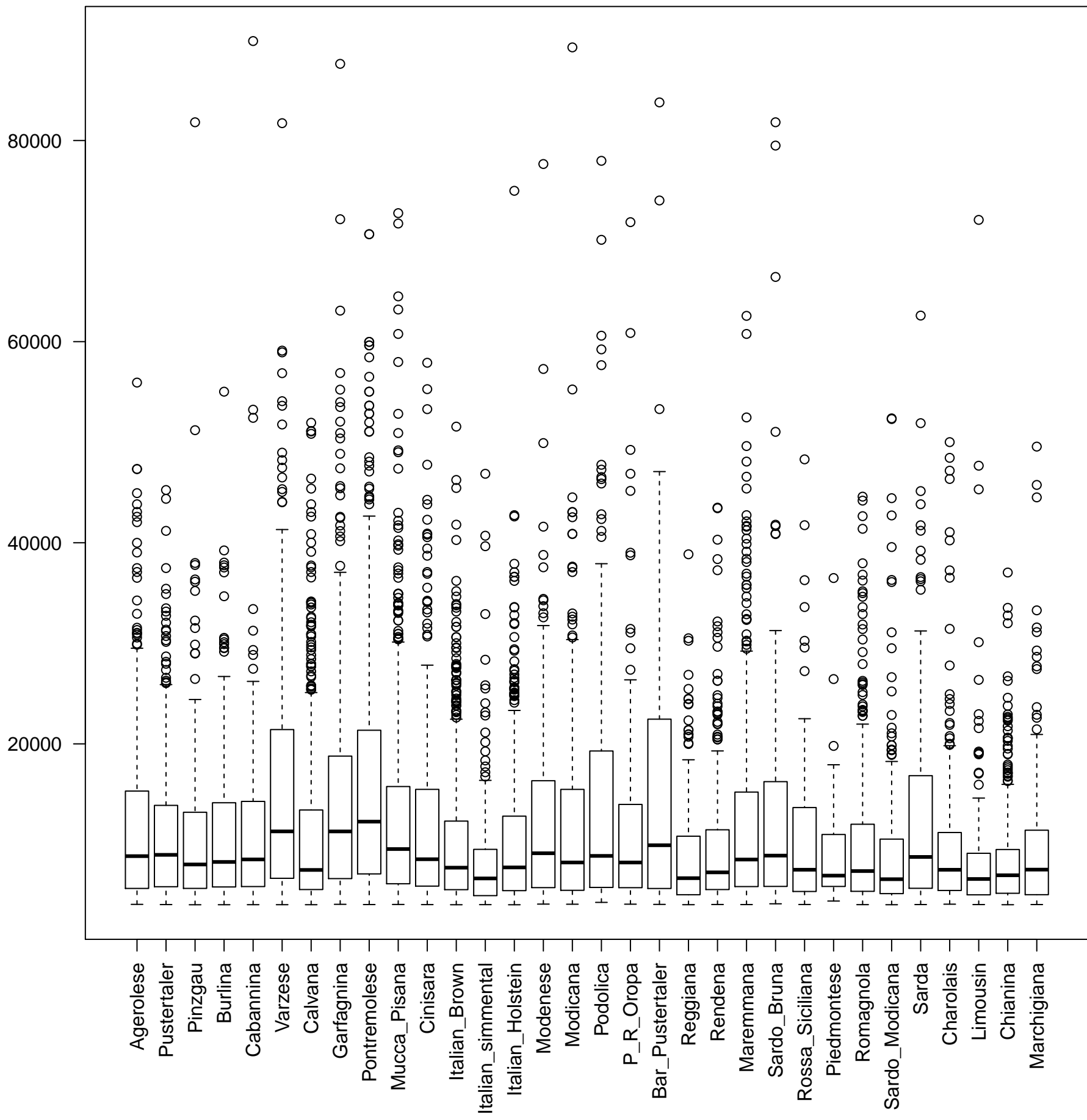

Supplement: Supplementary file 6 — Additional file 6: Figure S3. Distribution of all ROH within breeds according to their size (kb). [file 12711_2018_406_MOESM6_ESM.pdf]

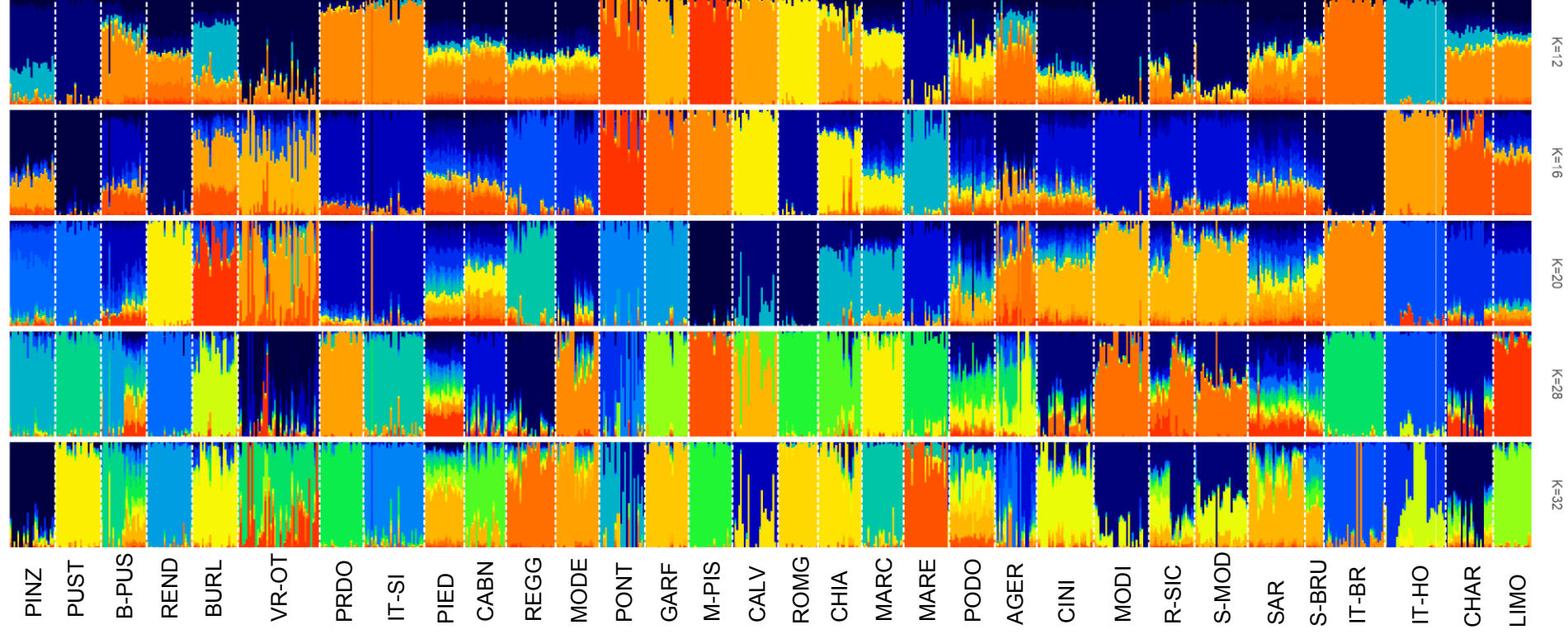

Supplement: Supplementary file 7 — Additional file 7: Figure S4. Model-based clustering of the estimated membership fractions of individuals from the 32 breeds analyzed in each of the K inferred clusters revealed by the ADMIXTURE software (K = 12, 16, 20, 28, 32). For a full definition of breeds see Table S1 (see Additional file 2: Table S1). [file 12711_2018_406_MOESM7_ESM.pdf]

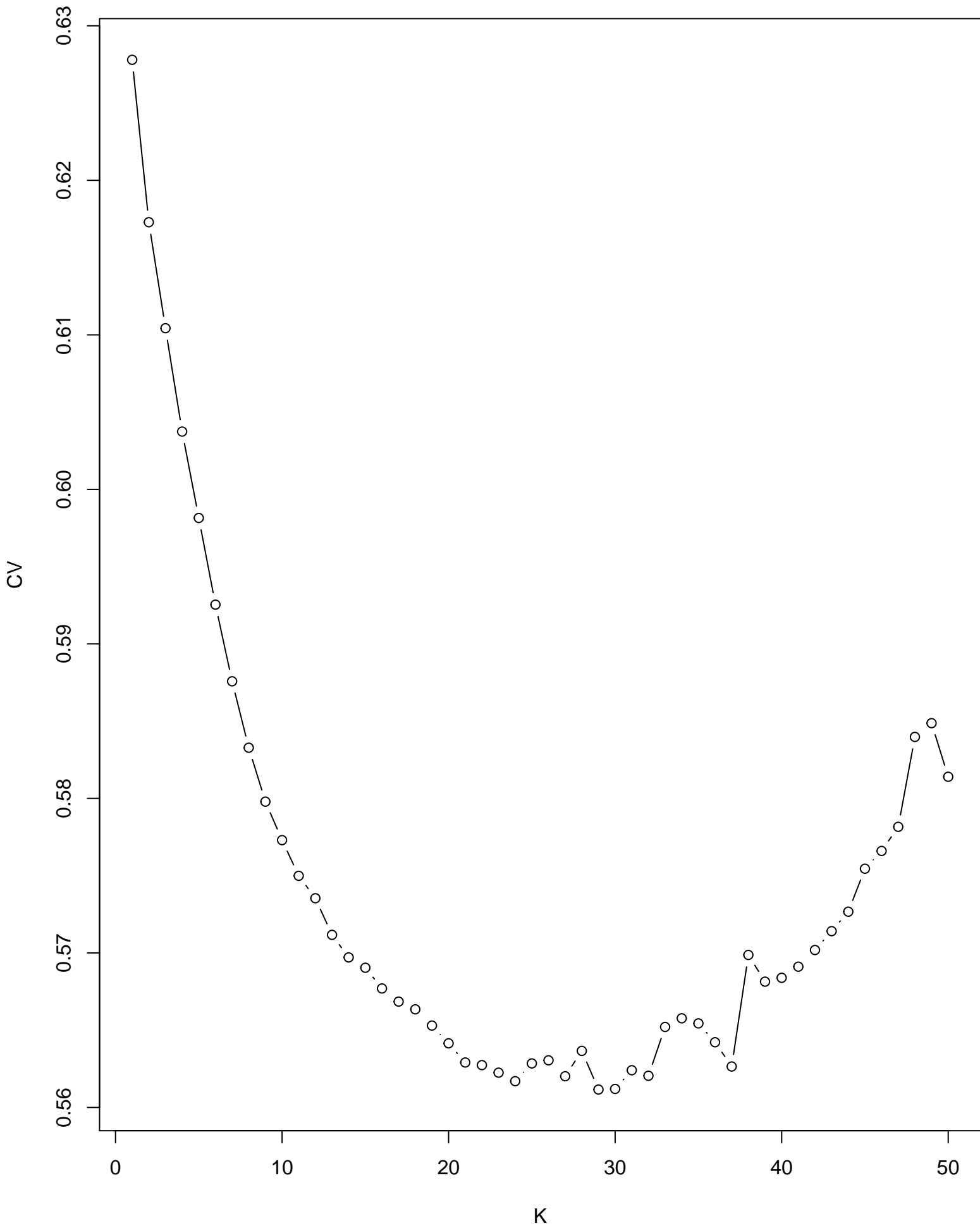

Supplement: Supplementary file 8 — Additional file 8: Figure S5. Cross-validation errors of admixture analysis at different K values. [file 12711_2018_406_MOESM8_ESM.pdf]

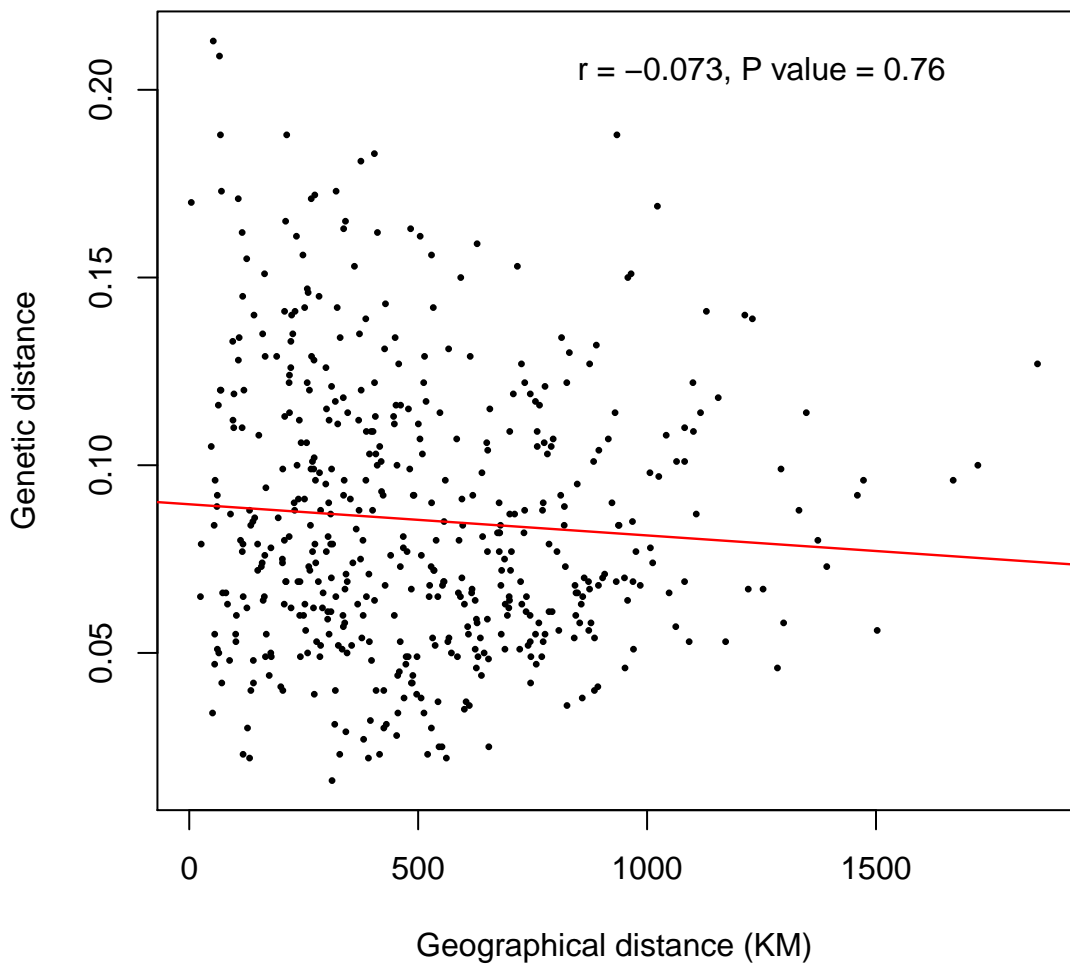

Supplement: Supplementary file 10 — Additional file 10: Figure S6. Scatter plot of correlations between genetic differentiation (FST) and geographical distances for all breeds. [file 12711_2018_406_MOESM10_ESM.pdf]

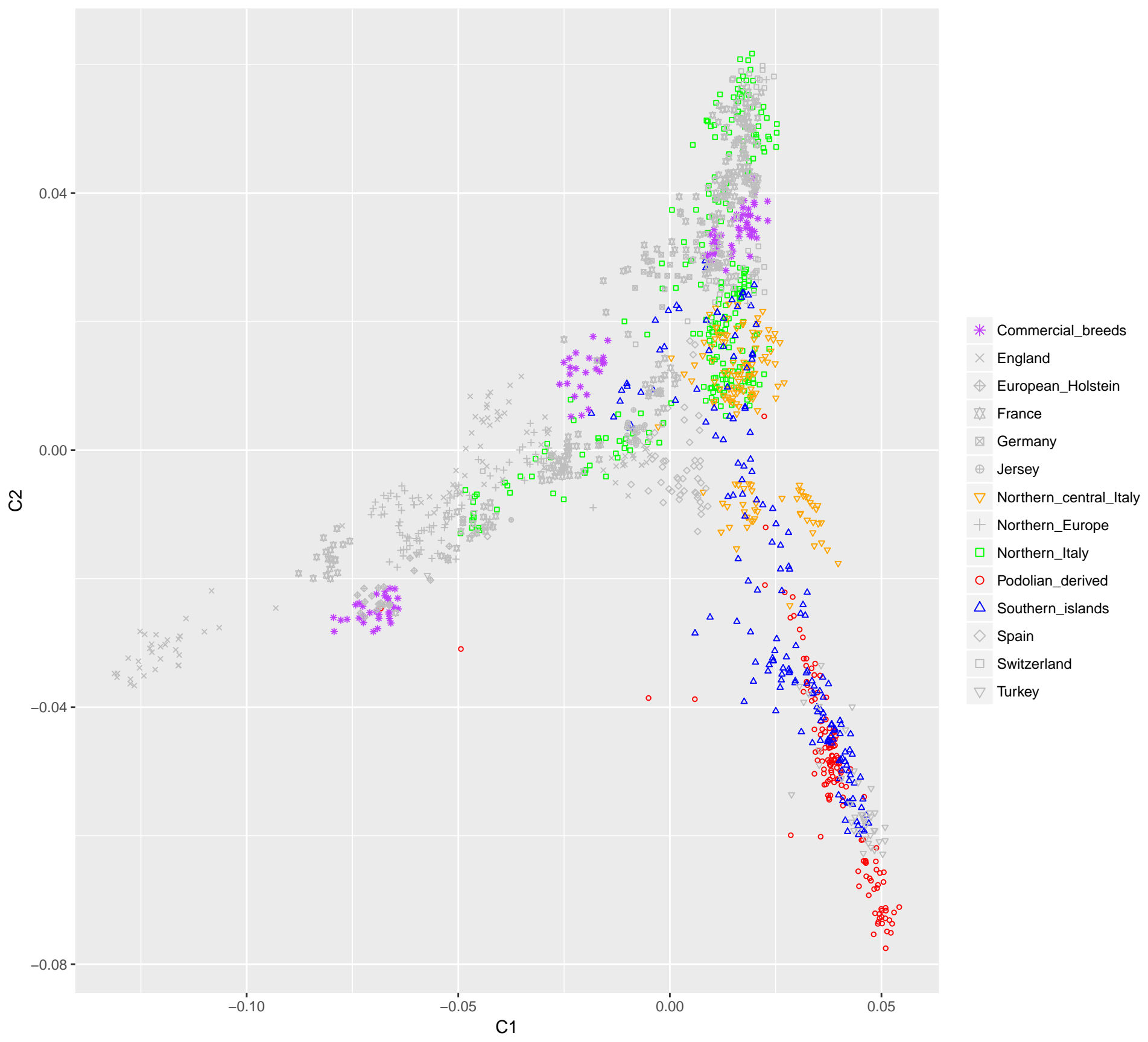

Supplement: Supplementary file 13 — Additional file 13: Figure S7. Genetic relationship defined with multidimensional scaling analysis between Italian and 62 European cattle breeds. The breeds were grouped according to their geographical origins and distributions. Northern_Europe (Belgian Blue, Dexter, Kerry, Lithuanian Light Grey, Lithuanian White Backed, Groningen Whitehead, Lakenvelder, Meuse-Rhine-Ijjsel, Norwegian Red, Finnish Ayrshire, Belted Galloway, Galloway, Angus, Scottish Highland, South Devon), England (Devon, Guernsey, Hereford, Longhorn, Lincoln Red, Milking Shorthorn, Red Poll, Beef Shorthorn, Sussex, Welsh Blach, White Park), Spain (Berrenda en Negro, Berrenda en Colorado, Cardena Andaluza, Menorquina, Pirenaica, Morucha, Mostrenca, Negra Andaluza, Toro de Lidia), France (Abondance, Aubrac, Blonde D’Aquitaine, Bretonne Black Pied, Gascon, Maine-Anjou, Maraichine, Montbeliard, Normande, French Red Pied, Salers, Tarine, Tarentaise, Vosgienne), Germany (Gelbvieh), Switzerland (Simmental, Simmentaler, Braunvieh, Ehringer), Turkey (Anatolian Black, Anatolian Southern Yellow, East Anatolian Red, South Anatolian Red, Turkish gray, Zavot). Italian breeds are colored according to their geographical distributions and are the same as those described in Fig. 2; the European breeds are colored in gray. [file 12711_2018_406_MOESM13_ESM.pdf]
