# Supplementary material for: Conservation status and historical relatedness of Italian cattle breeds
Source: Genet Sel Evol. 2018 Jun 26;50:35. doi: 10.1186/s12711-018-0406-x (PMC6019226; doi:10.1186/s12711-018-0406-x)
Supplement: Supplementary file 4 — Additional file 4: Table S2. Pearson correlation coefficients between genetic diversity indices. Observed (Ho) and expected (He) heterozygosity, average minor allele frequency (MAF), inbreeding coefficient (FHOM), contemporary effective population size (cNe), mean ROH-based inbreeding coefficient (FROH>4Mb) and recent and historical Ne estimated 13 (Ne13), 20 (Ne_20 and 80 (Ne_80) generations ago. (* p- value < 0.001). [file 12711_2018_406_MOESM4_ESM.docx]

|  | **Ho** | **He** | **MAF** | **F_HOM_** | **cNe** | **F_ROH>4Mb_** | **Ne_13** | **Ne_20** | **Ne_80** |
| --- | --- | --- | --- | --- | --- | --- | --- | --- | --- |
| **Ho** | - | 0.9393* | 0.9436* | -0.9998* | 0.1437 | -0.9202* | 0.6750* | 0.6911* | 0.7086* |
| **He** |  |  | 0.9991* | -0.9393* | 0.1562 | -0.8897* | 0.7939* | 0.8041* | 0.8133* |
| **MAF** |  |  |  | -0.9438* | 0.1505 | -0.8850* | 0.7854* | 0.7969* | 0.8096* |
| **F_HOM_** |  |  |  |  | -0.1426 | 0.9180* | -0.6723* | -0.6880* | -0.7055* |
| **cNe** |  |  |  |  |  | -0.2217 | -0.0204 | 0.0017 | 0.0049 |
| **F_ROH>4Mb_** |  |  |  |  |  |  | -0.7477* | -0.7567* | -0.7475* |
| **Ne_13** |  |  |  |  |  |  |  | 0.9966* | 0.9687* |
| **Ne_20** |  |  |  |  |  |  |  |  | 0.9846* |
| **Ne_80** |  |  |  |  |  |  |  |  | - |

**Additional File 4: Table S2.** Pearson correlation coefficients among genetic diversity indices.

Observed (Ho) and expected (He) heterozygosity, average minor allele frequency (MAF), inbreeding coefficient (F_HOM_), contemporary effective population size (cNe), mean ROH-based inbreeding coefficient (F_ROH>4Mb_) and recent and historical N_e_ estimated 13 (Ne13), 20 (Ne_20 and 80 (Ne_80) generations ago.

* p- value < 0.001
